# A GPU-Accelerated MRI Simulator for Synthetic Image Generation

**DOI:** 10.3390/jimaging12100477

**Published:** 2026-10-01

**Authors:** Riccardo Ferrero, Marta Vicentini, Elizabeth Cooke, Cormac McGrath, Aaron McCann, Amy McDowell, Nick Zafeiropoulos, Paul Tofts, Matt G. Hall, Alessandra Manzin

**Affiliations:** 1 Istituto Nazionale di Ricerca Metrologica (INRiM), 10135 Torino, Italy; m.vicentini@inrim.it (M.V.); a.manzin@inrim.it (A.M.); 2 National Physical Laboratory (NPL), Teddington TW11 0LW, UKmatt.hall@npl.co.uk (M.G.H.); 3 Forster Green Hospital, Belfast Health and Social Care Trust, Belfast BT8 6GR, UK; cormac.mcgrath@belfasttrust.hscni.net (C.M.); aaron.mccann@belfasttrust.hscni.net (A.M.); 4 University College London, London WC1E 6BT, UK; a.mcdowell@ucl.ac.uk (A.M.); nick.zafeiropoulos.11@ucl.ac.uk (N.Z.); 5 Brighton and Sussex Medical School, Brighton BN1 9PX, UK; bsms@paul-tofts.org.uk

**Keywords:** magnetic resonance imaging, diagnostic imaging, bloch equation solver, synthetic images, in silico modelling, digital phantoms

## Abstract

Magnetic Resonance Imaging (MRI) is an imaging technique that provides detailed structural and functional information about organs and tissues. To support the validation of MRI measurement techniques and provide a tool for scanner benchmarking, we develop an MRI simulator exploiting graphics processing units (GPUs) for computational acceleration. The GPU-Accelerated MRI Simulator (GAMS) enables us to generate synthetic images with high-resolution detail for quantitative estimation of relaxation times. GAMS is designed to reproduce the entire MRI image acquisition pipeline, including the definition of the MRI pulse sequence in a standard Pulseq format, the input of the digital phantom and its voxelization, the time integration of the Bloch equation at voxel level, and the processing of the derived k-space for image synthesis. The Bloch equation solver is implemented in CUDA Fortran, using GPUs to increase computational efficiency. The solver accuracy is tested in simple cases by comparison with analytical solutions and with the open-source Jülich Extensible MRI Simulator (JEMRIS). GAMS is applied to an inverse reconstruction problem to estimate the relaxation properties of a calibration phantom, starting with synthetic data. Then, it is used to reproduce the imaging process of a high-resolution digital brain phantom, generating realistic images under diverse MRI pulse sequences, which enable differentiation of tissues based on relaxation properties and/or proton density. The results demonstrate GAMS’s ability to generate synthetic images of complex anatomical structures comparable to those acquired with real MRI scanners and to reliably quantify sample relaxation properties. The simulator is also characterized by high computational efficiency, extensibility for multi-GPU implementation, and cross-platform compatibility. These features make GAMS a valuable tool for the metrological assessment of quantitative MRI (qMRI) and the testing of new pulse sequences for qMRI technique development.

## 1. Introduction

Magnetic Resonance Imaging (MRI) is a widely adopted non-invasive diagnostic imaging technique, which finds extensive application in clinical and research settings due to its unparalleled ability to provide detailed structural and functional information on different organs in healthy and pathological conditions [1,2,3,4]. Typically, the acquisition of clinical MRI images is susceptible to differences in pulse sequence protocol and to the type of scanner used, making the comparison of data obtained in large-scale trials very complex. Quantitative MRI (qMRI) offers a way to improve this process, since measurements of physical quantities can be independently verified, and the performance of individual scanners can be benchmarked and quantified [5,6,7,8,9]. However, the testing and validation of MRI-based measurement processes need reference data and methodologies that allow different error sources to be investigated separately. Simulation-based approaches can support the validation phase, since they enable the reproduction of the imaging steps, as a function of the acquisition parameters, e.g., static magnetic field amplitude, type of pulse sequence [10,11,12], and of the material properties of the imaged test object or phantom. Synthetic images can be directly compared with measured ones under simple, controlled experimental conditions reproducible in the laboratory. Moreover, they can be exploited in complex scenarios, e.g., guiding the investigation of image artifacts due to implants [13,14], high-resolution quantitative mapping of tissue relaxation properties [15], extracting radiomic features [16], and providing synthetic data for the development of data-driven deep learning image reconstruction techniques for qMRI [17].

First, the generation of synthetic images for MRI needs the availability of detailed digital phantoms discretized in volume elements (voxels) with associated material properties, i.e., spin-lattice relaxation time $T_{1}$, spin-spin relaxation time $T_{2}$, and proton density PD [18]. These test objects can be digital reproductions of calibration phantoms, like the ones developed by the U.S. National Institute of Standards and Technology (NIST) [5,19,20], or digital reconstructions from medical images of anatomical structures and organs, like the brain [21,22,23] and breast [24,25]. Second, MRI simulation-based approaches need the availability of accurate and efficient computational models that implement the Bloch equation to describe nuclear spin relaxation processes [26].

First attempts to solve the Bloch equation focused on analytical methodologies, applied under simplifying conditions to enable the understanding of fundamental principles [27,28]. As MRI technology evolved, in order to encompass different acquisition strategies, scanner configurations, and complex spatial distributions of tissue properties, numerical solvers integrating the Bloch equation started to be developed [29]. Currently, the Jülich Extensible MRI Simulator (JEMRIS) [30] is one of the most notable examples of open-source MRI simulators, providing a multi-purpose tool for investigating many aspects of MRI physics. Other numerical codes have been developed to solve inverse problems aimed at tissue magnetic susceptibility mapping [31] as well as to include specific phenomena involved in MRI, such as molecular diffusion in biological tissues and motion-induced artifacts [32,33].

Thanks to the highly parallel nature of the problem and the widespread adoption of graphics processing units (GPUs) as computational accelerators, progress has been made with the implementation of GPU-based parallel solvers [34,35,36,37] and the GPU adaptation of open-source simulators, like JEMRIS [38]. Recently, deep learning-based solvers, e.g., Simu-Net [39], have also been implemented to accelerate Bloch simulation in combination with GPU use. In this framework, we have developed a GPU-Accelerated MRI Simulator (GAMS), able to run in a CUDA-compatible GPU-based environment, adapting previous micromagnetic codes created for the simulation of magnetization relaxation processes in magnetic nanomaterials [40,41,42]. The aim of this solver is to produce synthetic images that can be used for the calibration of MRI scanners and the analysis of the sensitivity of image generation to magnetic field perturbations and uncertainties in the relaxation parameters, in an effort to provide a metrological framework for qMRI’s increasing adoption [43]. GAMS is designed to enable the full integration of the Bloch equation in a 3D domain and the accurate reproduction of the complete image generation process. More specifically, it allows the computation of classical spin dynamics under the effect of contrast-generating MRI pulse sequences, including full 3D image generation starting from voxel-based digital phantoms. Spin excitation and spatial encoding field pulses are inputted according to the Python Package for MRI Pulse Sequence Design (PyPulseq) [44,45]; the computed magnetization components are then acquired at well-defined time intervals to produce the MRI signal, from which the synthetic image is generated.

In this work, GAMS is described in Section 2 and benchmarked in Section 3 in different test cases, where the reference solution is evaluated analytically or by means of the open-source simulator JEMRIS [30]. Computational performance is also evaluated in comparison to JEMRIS and its GPU-accelerated version. In Section 4, we evaluate the ability of GAMS to reproduce qMRI experiments by simulating two standard relaxometry techniques for the recovery of $T_{1}$ and $T_{2}$. Then, we test its capability to produce realistic image contrasts by comparing its outputs against real MR images acquired on a test phantom, under similar relaxometry experimental conditions. An experiment that includes the effects of static magnetic field distortions is also considered. Finally, GAMS is used for the generation of synthetic images of a human brain digital phantom, comparing the results obtained by applying different MRI pulse sequences in terms of tissue contrast appearance.

## 2. Materials and Methods

**Figure 1:**
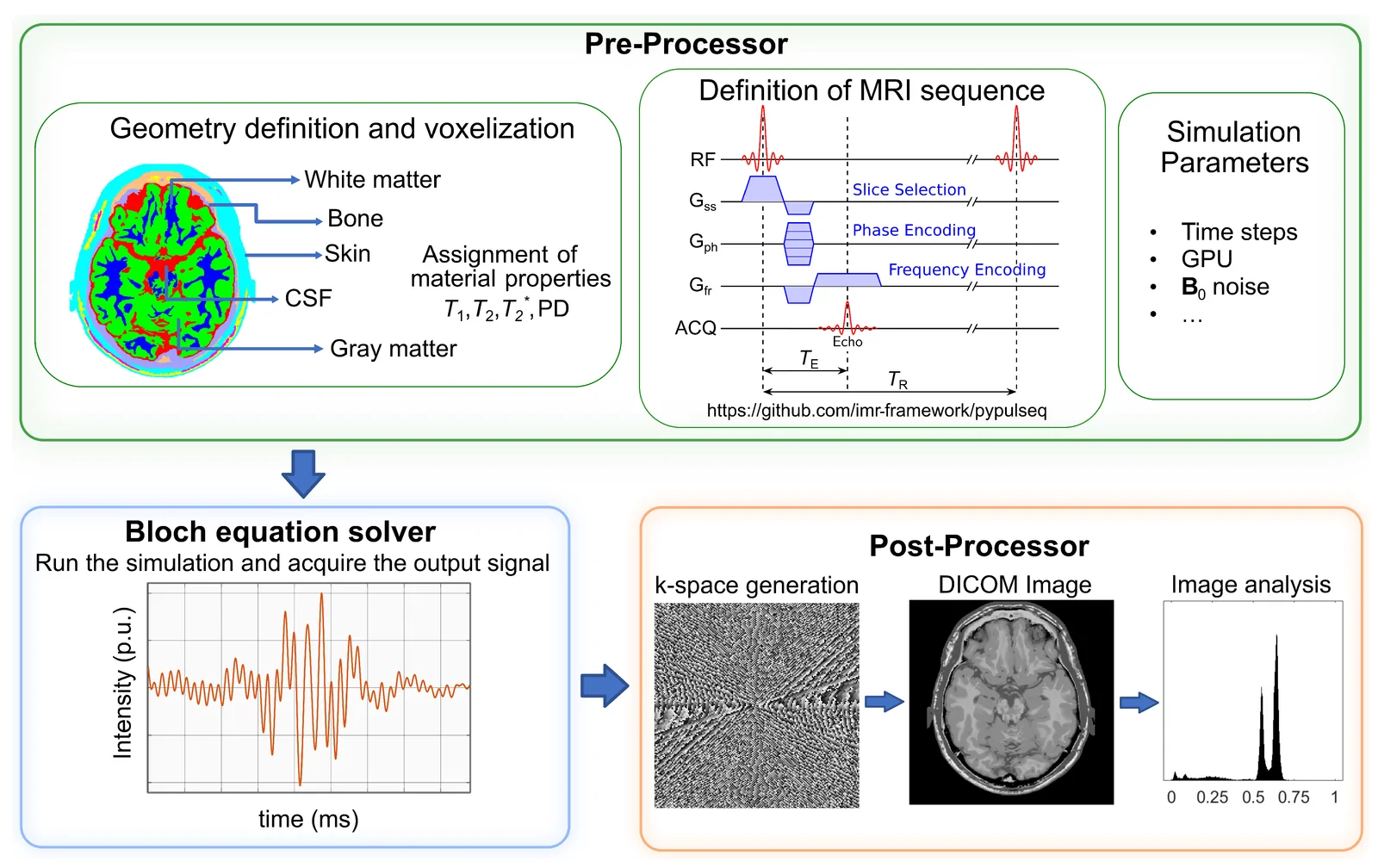
Schematic of the pipeline implemented in GAMS for the generation of synthetic MRI images, comprising (i) the input of phantom geometrical and material properties, (ii) the selection of MRI pulse sequence and simulation parameters, (iii) the Bloch equation-based simulation, (iv) the signal acquisition with k-space generation and (v) the post-processing of k-space for DICOM image generation. In the sequence schematic, $G_{\mathrm{ss}}$, $G_{\mathrm{ph}}$ and $G_{\mathrm{fr}}$ are the gradients along slice-selection, phase-encoding and frequency-encoding directions, respectively; ACQ indicates the data acquisition period.

GAMS enables us to simulate the full pipeline for the generation of synthetic MRI images, including the design and input of the MRI pulse sequence, the definition of the 3D computational domain (digital phantom), its discretization in voxels, the time integration of the Bloch equation in each voxel, and the numerical signal processing. GAMS includes three components: (1) the pre-processor, for the input of geometrical, physical, excitation and acquisition parameters; (2) the GPU-accelerated Bloch equation solver, for the production of the raw-data signal by means of in-house developed CUDA-Fortran routines; and (3) the post-processor, for the processing of the generated signal and its conversion into a Digital Imaging and Communications in Medicine (DICOM) file with associated metadata. The full pipeline for the synthetic MRI image generation is schematically described in Figure 1.

### 2.1. Pre-Processor

The pre-processor allows us to generate the input files for the Bloch equation solver, describing the geometrical and physical properties of the digital phantom, and the excitation and acquisition parameters necessary to produce the MRI images. The required data are listed below.

- *Phantom geometrical properties*: The phantom is described by means of a mesh file, which provides the 3D structure as a regular grid of voxels with associated material codes. The grid can be derived from any CAD (Computer-aided design) model by converting the closed surface shells at the interface between materials to discretized volumes.
- *Phantom material properties*: For each material code, a set of relevant physical properties is assigned, comprising the relaxation times ($T_{1}$, $T_{2}$) and the proton density (PD), expressed in percentage units of the proton concentration in water.
- *Scan and sequence parameters*: These include the amplitude of the static magnetic field $B_{0}$ with associated spatial perturbations, the scan slice orientation, the type of MRI pulse sequence and its specifications, e.g., the radiofrequency (RF) field $B_{1}$, applied perpendicularly to $B_{0}$, the echo time $T_{E}$, the repetition time $T_{R}$, the inversion time $T_{I}$, and the gradient intensities for slice selection, phase encoding and frequency encoding.
- *Integration parameters*: These include the time-steps used to solve the Bloch equation in each voxel during the excitation and relaxation intervals, respectively.

For the sake of interoperability and ease of use, we have employed the open-source framework Pulseq for the design of the MRI pulse sequences, adopting version 1.3.1 for the file format [44]. In this way, the sequence file can be easily generated with JEMRIS (version 2.9.1) or with the PyPulseq Python package (version 1.3.1) [45]. The sequence-generating Python scripts used to produce the results shown in this work are available in the Scripts section of the Supplementary Materials.

### 2.2. GPU-Accelerated Bloch Equation Solver

The MRI signal is generated by numerically solving the phenomenological Bloch equation, which is normally used to model nuclear spin dynamics under the effect of external magnetic fields [26]. A rotating frame $\left(x_{\mathrm{rot}},y_{\mathrm{rot}},z_{\mathrm{rot}}\right)$ is considered, with the axis of rotation $z_{\mathrm{rot}}$ parallel to the static field direction (*z*-axis) and rotating with an angular frequency $\omega_{0}=\gamma B_{0}$, corresponding to the Larmor frequency of protons under the effect of $B_{0}$ (γ is the proton gyromagnetic ratio). In this frame, the Bloch equation assumes the following expression:

(1) $$\frac{dM_{\mathrm{rot}}}{dt}=\gamma M_{\mathrm{rot}}\times B_{\mathrm{eff}}-\frac{M_{z_{\mathrm{rot}}}-M_{0}}{T_{1}}{\hat{z}}_{\mathrm{rot}}-\frac{M_{x_{\mathrm{rot}}}{\hat{x}}_{\mathrm{rot}}+M_{y_{\mathrm{rot}}}{\hat{y}}_{\mathrm{rot}}}{T_{2}}$$

where $M_{\mathrm{rot}}=\left(M_{x_{\mathrm{rot}}},M_{y_{\mathrm{rot}}},M_{z_{\mathrm{rot}}}\right)$ is the macroscopic nuclear magnetization vector within a single voxel and $B_{\mathrm{eff}}$ is the effective field experienced by the magnetization. $M_{0}$ is the longitudinal equilibrium magnetization, described as

(2) $$M_{0}=\frac{\gamma^{2}\hbar^{2}N_{s}}{4k_{B}T_{a}}B_{0},$$

where *ℏ* is the reduced Planck constant, $k_{B}$ is the Boltzmann constant, $T_{a}$ is the ambient temperature, and $N_{s}$ is the number of protons per unit volume or spin (proton) density. In our implementation, we adopt the usual expression of the proton density in percentage units (p.u.) of the proton density in water $N_{w}$, i.e., $\mathrm{PD}=N_{s}/N_{w}$.

The effective magnetic field in Equation (1) is expressed as

(3) $$B_{\mathrm{eff}}=B_{1}\left(t\right)+\left[G\left(t\right)\cdot r\right]{\hat{z}}_{\mathrm{rot}}+B_{0}-\frac{\omega_{0}}{\gamma }{\hat{z}}_{\mathrm{rot}}+\left[\frac{\Delta \omega (t)}{\gamma }+\Delta B_{0}\right]{\hat{z}}_{\mathrm{rot}}$$

The first term in Equation (3), $B_{1}\left(t\right)$, corresponds to the circularly polarized RF contrast-generating pulse, applied perpendicularly to $B_{0}$. We tune the RF pulse to the Larmor frequency and set the initial phase angle at zero, to conveniently have only one field component different from zero, i.e., $B_{1}=B_{1}\left(t\right){\hat{x}}_{\mathrm{rot}}$. The second term in Equation (3), $\left[G(t)\cdot r\right]{\hat{z}}_{\mathrm{rot}}$, contains the frequency-encoding (along *x*), phase-encoding (along *y*) and slice-selection (along *z*) gradient components, and is responsible for a local shift of the spin angular frequency, which depends on the voxel vector position **r** and gradient vector G(t). The third term in Equation (3) is the static field in the rotating frame, $B_{0}=B_{0}{\hat{z}}_{\mathrm{rot}}$, while the fourth term, $-\left(\omega_{0}/\gamma \right){\hat{z}}_{\mathrm{rot}}$, is the apparent field that emerges when passing from the laboratory (stationary) frame to the rotating one. The fifth term describes possible distortion effects, which comprise off-resonance distortion contributions due to chemical shifts, $\left[\Delta \omega (t)/\gamma \right]{\hat{z}}_{\mathrm{rot}}$, as well as local inhomogeneities of the static magnetic field, $\Delta B_{0}{\hat{z}}_{\mathrm{rot}}$. In GAMS, the latter can be introduced as a map of perturbations at voxel level (e.g., derived from static field measurement), or as random fluctuations in space. This contribution is responsible for an extra dephasing effect causing a faster magnetization decay and thus a signal loss [46].

The time evolution of the different magnetic field terms described above is encoded into event blocks according to the Pulseq library specifications [44,45]. These comprise excitation blocks, encoding blocks, and delay blocks, which refer respectively to the application of the RF pulse, the magnetic field gradients and the static field; readout blocks are also considered to parametrize signal acquisition. The Bloch equation is independently solved in each voxel, exploiting parallelization in a GPU-based computational framework. In each voxel, a 4th-order Runge–Kutta time-integration scheme is used to solve Equation (1), updating at each time instant the effective magnetic field according to Equation (3), using double-precision arithmetic. Time-steps in the order of $10^{-6}$ s are selected during the application of the time-varying magnetic fields, while time-steps around $10^{-3}$ s are used during the delay blocks, to speed up the computation. Along the readout blocks, at precise time intervals specified by the selected MRI pulse sequence, the two transverse magnetization components, $M_{x_{\mathrm{rot}}}$ and $M_{y_{\mathrm{rot}}}$, are integrated over the phantom volume to obtain the MRI signal, which is then stored for the post-processing step.

### 2.3. Post-Processor

The solver can reproduce a variety of k-space trajectories (Cartesian, radial, spiral), since it can import any gradient shape available in Pulseq format. The acquired signal is mapped to the k-space according to the sequence file instructions. For Cartesian (line-by-line) sampling, which is the most straightforward form of MRI readout [47], the frequency and phase encoding steps are evenly spaced on a regular grid; thus, the final grayscale image can be obtained directly by applying the inverse Fast Fourier Transform (FFT) algorithm. In spiral and radial trajectories, the k-space is not sampled uniformly, with more sampling points acquired toward the center of the k-space and fewer moving outward, requiring that the signal is weighted accordingly. The image is reconstructed using a non-uniform FFT algorithm (nuFFT) [48,49]. The output is stored as a DICOM file together with the associated metadata file, which contains information on the selected MRI pulse sequence and on the parameters used for the k-space derivation, e.g., sequence name, trajectory type, echo time $T_{E}$, repetition time $T_{R}$, inversion time $T_{I}$, flip angle, echo train length, data acquisition period (ACQ), number of slices, number of steps for phase encoding and frequency encoding, and FOV (Field of View). Optionally, at each timestep, the individual spin dynamics can be exported in raw single-precision format.

## 3. Accuracy and Computational Performance Analysis

We test the accuracy of the Bloch equation solver implemented in GAMS by performing two numerical experiments. First, the simulation results are compared to the analytical solution for the free induction decay (FID) signal. Second, the synthetic images generated by applying the entire developed pipeline are compared to the ones obtained with the open-source MRI simulator JEMRIS [30], considering two test objects. We also examine GAMS in terms of computational efficiency with respect to JEMRIS.

For all the simulations reported here, we consider the following scan parameters: an amplitude of 1.5 T for the static magnetic field; a maximum gradient amplitude of 32 $\mathrm{mT}\;m^{-1}$; and a maximum gradient slew rate of 130 $T\;m^{-1}s^{-1}$.

### 3.1. Comparison to Analytical Solution for Free Induction Decay

**Figure 2:**
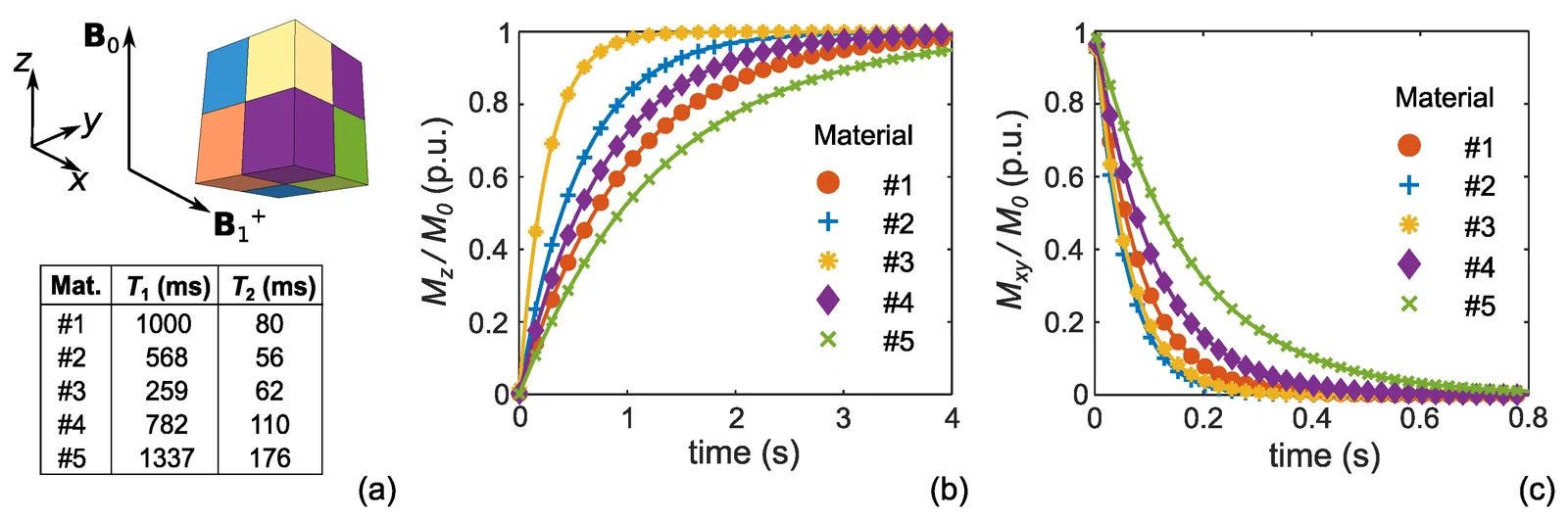
(**a**) Schematic of the cubic phantom used for the FID experiment and table reporting the $T_{1}$ and $T_{2}$ values of the five constituent materials. (**b**) Time evolution of the magnetization calculated in five voxels made of different materials: comparison between numerical results (markers) and analytical solution (continuous lines) for (**b**) longitudinal and (**c**) transverse components.

The Bloch equation solver is first used to calculate the time evolution of the magnetization after the application of a single $90^{\circ }$-FID pulse. The solution for this problem is known analytically and can be used as a reference to validate the simulation results [26]. The considered test object is a 2×2×2 voxel-based cube, which contains five materials distributed among the voxels and characterized by different values of longitudinal $T_{1}$ and transverse $T_{2}$ relaxation times, varying within the ranges 259–1337 ms and 56–176 ms, respectively (Figure 2a). We apply a $90^{\circ }$ pulse, without any slice-selective gradient, to flip the magnetization of the whole phantom perpendicularly to its initial equilibrium state, parallel to the static field. Following the pulse, the magnetization relaxes under the effect of only the static field toward the initial equilibrium. In each voxel, the exact solution can be expressed as:

(4) $$\left\{\begin{array}{cc} M_{z}\left(t\right) & =M_{0}\left(1-e^{-t/T_{1}}\right)\\ M_{xy}\left(t\right) & =M_{0}e^{-t/T_{2}} \end{array}\right.$$

The normalized magnetization components parallel and perpendicular to the static field, calculated for each material, are reported in Figure 2b and Figure 2c, respectively, together with the corresponding analytical solutions. The numerical results, which are obtained by fixing the time-step to 5 ms, are in very good agreement with the exact solutions for the entire range of variation of parameters $T_{1}$ and $T_{2}$. Let $M_{k}\left(t_{i}\right)$ be the magnetization component along the longitudinal or transverse direction for the generic *k*-th material, computed at time instant $t_{i}$, and let ${\hat{M}}_{k}\left(t_{i}\right)$ be the corresponding analytical solution. The maximum absolute error

(5) $$\varepsilon_{\max,k}=\max_{k,i}\left|\frac{M_{k}\left(t_{i}\right)-{\hat{M}}_{k}\left(t_{i}\right)}{M_{0}}\right|$$

is always lower than $6\cdot 10^{-8}$. This error decreases to $1\cdot 10^{-8}$ when the time-step is reduced to 0.1 ms, while a further diminution of the time-step does not improve the numerical accuracy.

### 3.2. Comparison with MRI Simulator JEMRIS

As a second test case, we compare the Bloch equation solver with the MRI simulator JEMRIS (version 2.9.1), which enables us to easily design MRI sequences and export them to the Pulseq format we have adopted [44,45]. Specifically, we generate DICOM images of two test objects using a Gradient Recalled Echo (GRE) pulse sequence, which consists of a series of RF pulses with a flip angle lower than $90^{\circ }$ and imaging gradients (see sequence schematic in Figure S1 of the Supplementary Materials). In the considered examples, the magnetization is flipped at a constant angle of $20^{\circ }$, the repetition time $T_{R}$ (between successive RF pulses) is fixed to 500 ms, and the echo time $T_{E}$ (between RF pulse and gradient echo formation) is set at 8 ms; no slice-select gradient is imposed. To minimize the differences that can be introduced by the two image reconstruction pipelines and maintain the image processing consistent between the two calculated signals, the raw data of the k-space extracted from JEMRIS-based simulations are processed with the same pipeline we have implemented for signal processing in GAMS.

The first test object is constituted by two concentric disks with diameters of 100 mm and 50 mm, respectively, and a thickness of 1 mm. For the inner disk $T_{1}=100$ ms and $T_{2}=50$ ms, while for the outer one, $T_{1}=200$ ms and $T_{2}=100$ ms; the proton density is the same in both disks and is assumed equal to the water proton density (1 p.u.). This test object is discretized with two spatial resolution meshes, having voxel sizes of 1×1×1 ${\mathrm{mm}}^{3}$ and 0.1×0.1×1 ${\mathrm{mm}}^{3}$, respectively. For the low-resolution case, we generate a 64×64 px image (each pixel corresponds to an area of 2×2 ${\mathrm{mm}}^{2}$), while for the high-resolution case, we generate a 256×256 px image (each pixel corresponds to an area of 0.5×0.5 ${\mathrm{mm}}^{2}$). The slice thickness is the same in the two cases and corresponds to the phantom height.

**Figure 3:**
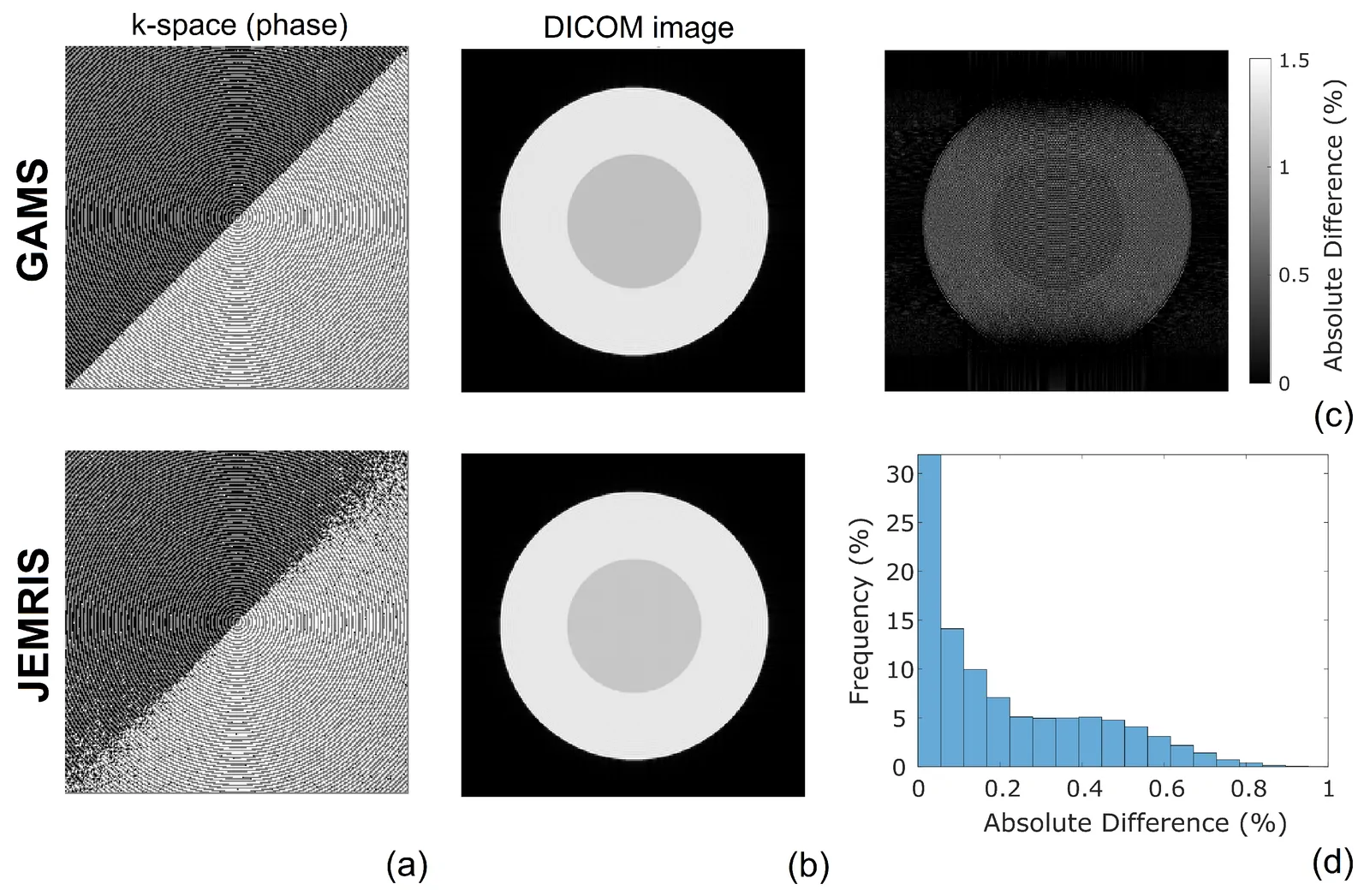
Comparison between the simulation results obtained with GAMS and JEMRIS, by applying a GRE sequence for the 256×256 px imaging of a two-concentric-disk-based phantom with 0.1 mm voxel size. (**a**) Maps of the k-space phase. (**b**) DICOM images. (**c**) Pixel-by-pixel map of the absolute difference in gray level intensity between the obtained images. (**d**) Frequency plot of the absolute difference at pixel level.

For the high-resolution case, the maps of the magnitude of the *k*-spaces extracted from GAMS- and JEMRIS-based simulations are very similar, while small discrepancies can be appreciated by comparing the relative phase maps (Figure 3a). In particular, the phase map generated with JEMRIS shows a less regular pattern, especially in the top-right and bottom-left corners of the map, where background noise appears. Nevertheless, the DICOM images obtained with GAMS and JEMRIS are practically indistinguishable (Figure 3b). The reconstructed magnitude images from both simulators are independently normalized to their respective maximum intensities, scaling the gray levels to a [0,1] range. All comparative metrics are calculated over the entire image FOV (including background pixels) to capture both the structural representation of the phantom and the background noise characteristics. The Structural Similarity Index Measure (SSIM) [50] is 0.997. To better analyze the discrepancies between the two generated images, we calculate the pixel-by-pixel map of the absolute difference in gray level intensity (Figure 3c) and the histogram illustrating the number of pixels versus the local image difference (Figure 3d). The same procedure is performed for the low-resolution case, whose results, which are reported in Figure S2 of the Supplementary Materials, are characterized by a higher level of discrepancies, confirmed by a slightly lower SSIM value of 0.985.

The two sets of images are characterized by a pixel-by-pixel maximum difference of 1.1% and 2.3% for the high- and low-resolution cases, respectively, with a corresponding Mean Absolute Difference $\mathrm{MAD}=\frac{1}{N}\sum_{n=1}^{N}\left|S_{n}^{\mathrm{GAMS}}-S_{n}^{\mathrm{JEMRIS}}\right|$ in signal gray level intensity *S* between the *N* pixels of 0.2% and 0.3%. The Root Mean Squared Error (RMSE) between the calculated signals is 0.3% and 0.4% for the high- and low-resolution cases, respectively.

**Figure 4:**
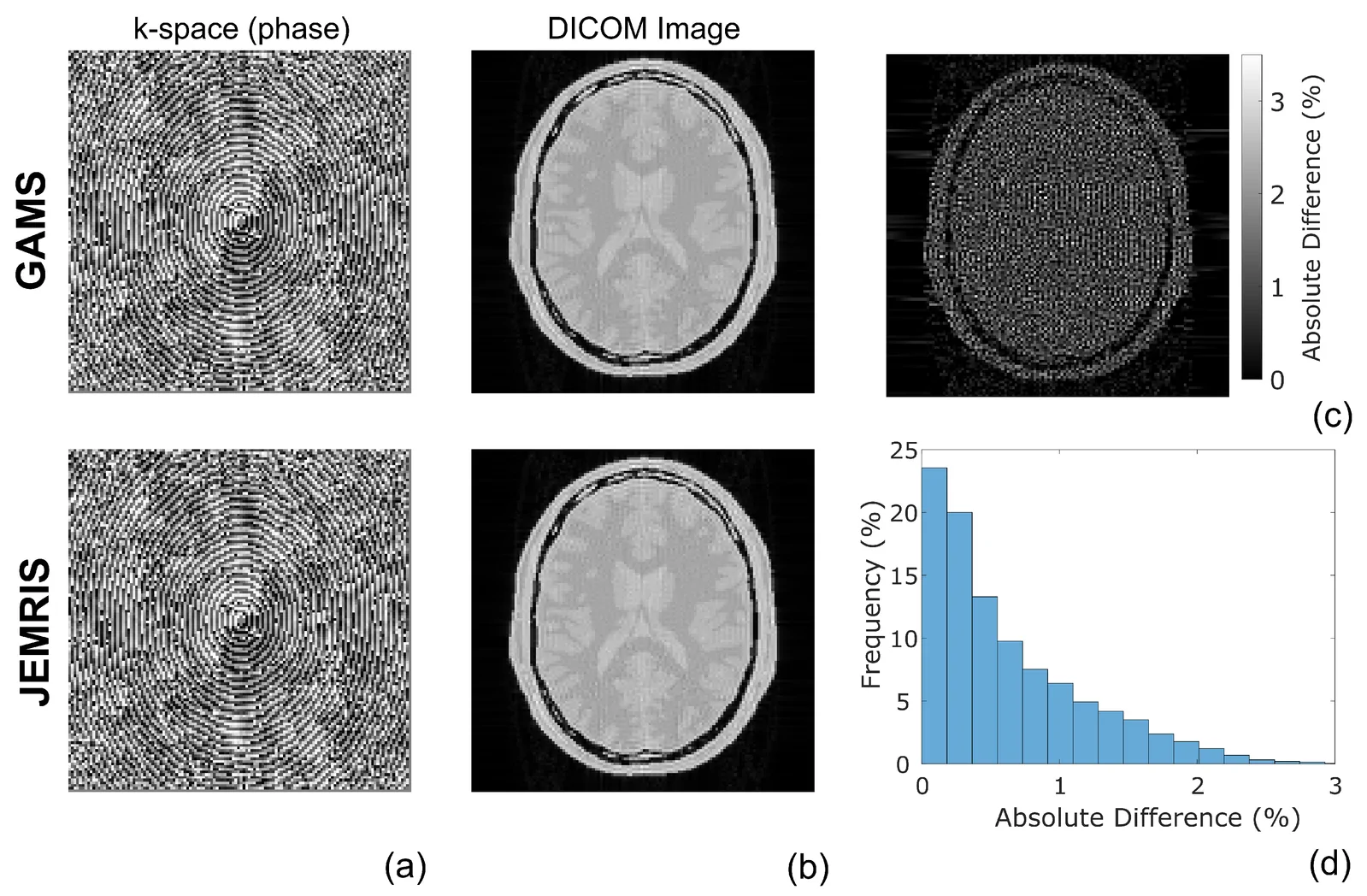
Comparison between the simulation results obtained with GAMS and JEMRIS by applying a GRE sequence for the 128×128 px imaging of a slice of a 3D brain model with 1 mm voxel size. (**a**) Maps of the k-space phase. (**b**) DICOM images. (**c**) Pixel-by-pixel map of the absolute difference in gray level intensity between the obtained images. (**d**) Frequency plot of the absolute difference at pixel level.

The second test object is constituted by a single slice of the 3D brain phantom available in the JEMRIS simulation framework [30], having dimensions of 180×216×1 ${\mathrm{mm}}^{3}$ and a voxel size of 1×1×1 ${\mathrm{mm}}^{3}$. The phantom is composed of seven materials, whose properties ($T_{1}$, $T_{2}$ and PD) are provided by JEMRIS and listed in Table S1 of the Supplementary Materials. We acquire an image with a resolution of 128×128 px, where each pixel corresponds to an area of 1.7×1.7 ${\mathrm{mm}}^{2}$. As shown in Figure 4a, possible discrepancies between the maps of the k-space phase extracted from GAMS- and JEMRIS-based simulations are hidden by the complex fine structure of the phantom. The two obtained DICOM images are qualitatively very similar (Figure 4b), with an SSIM value of 0.948. However, the pixel-by-pixel map of the absolute difference in gray level intensity (Figure 4c) and the associated histogram (Figure 4d) highlight a maximum discrepancy in the order of 3.5% and a MAD of 0.6%; the RMSE between the acquired signals is 0.7%. These values are slightly larger than the ones found for the previous test object due to the increased complexity in both material composition and geometrical structure.

### 3.3. Analysis of Computational Efficiency

To evaluate the computational efficiency of GAMS in a practical scenario, we perform a system-level throughput benchmark comparing it against the established CPU-based JEMRIS simulator (version 2.9.1) [30] and its recently introduced GPU-accelerated variant (version 2.9.2) [38]. The performance test is carried out using a 2D numerical test object consisting of two concentric disks, varying the phantom voxel size from 1×1×1 ${\mathrm{mm}}^{3}$ to 0.1×0.1×1 ${\mathrm{mm}}^{3}$ (the corresponding number of phantom voxels $N_{v}$ increases from $10^{4}$ to $10^{8}$).

**Figure 5:**
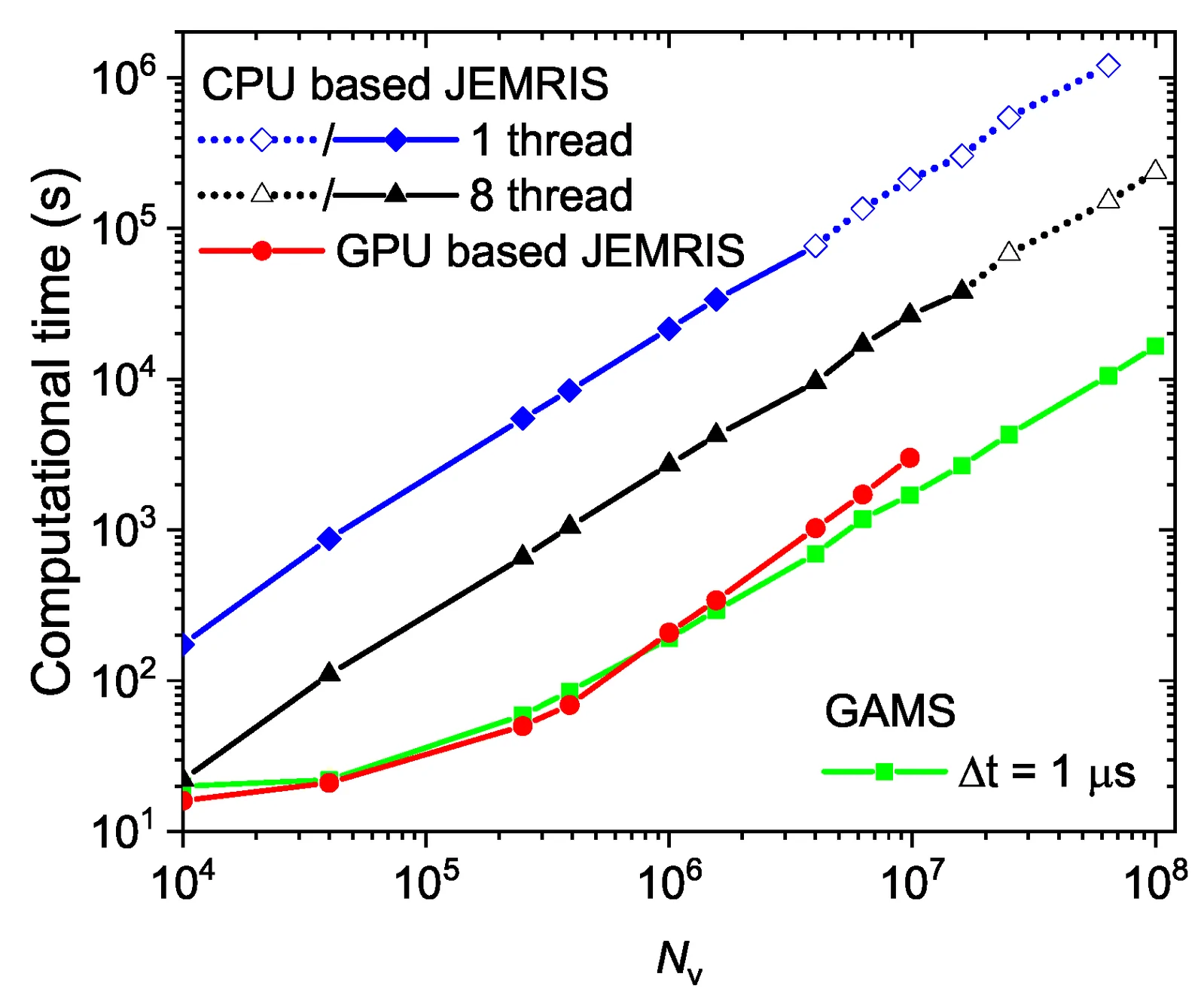
Comparison between the computational times of GAMS, CPU-based JEMRIS, and GPU-based JEMRIS as a function of the number of voxels $N_{v}$ used for the test object discretization. A GRE sequence with an acquisition resolution of 64×64 px is chosen for the analysis. GAMS is tested fixing the time-step Δt to 1 μs. Both CPU-based JEMRIS and GPU-based JEMRIS utilize variable time-stepping solvers; CPU-based JEMRIS is executed in single-thread and 8-thread parallel modes, whereas GPU-based JEMRIS is executed on the GPU. The open symbols indicate extrapolated computational time values. To encompass all the components of the MRI acquisition pipeline with a sufficiently short time window, we select a GRE sequence with the same parameters as in Section 3.2 and an image resolution of 64×64 px.

CPU-based JEMRIS and GPU-based JEMRIS simulations are set up via the graphical user interface and later run independently via the command line. All simulations are executed on a workstation equipped with an Intel® Xeon® w9-3475X processor (Santa Clara, CA, USA) and an NVIDIA RTX A6000 GPU (Santa Clara, CA, USA). The simulators are configured as follows: CPU-based JEMRIS using its standard variable time-stepping solver in both single-thread and 8-thread parallel modes; GPU-based JEMRIS is executed on the GPU utilizing its native variable time-stepping solver; and GAMS is executed on the GPU using the fixed-step RK4 solver with time-step Δt=1μs. For the test, we employ the GRE sequence described in Section 3.2 with a k-space matrix of size 64×64. The computational time for the four considered cases is reported in Figure 5 as a function of $N_{v}$; for the simulations performed with CPU-based JEMRIS, the data for the higher values of $N_{v}$ are ideally extrapolated, assuming a linear relationship (open symbols). Parallelized JEMRIS demonstrates an 8-fold improvement over the single-thread simulation across the entire tested voxel range. For $N_{v}=10^{4}$, the GPU is underutilized and the performance of GAMS with Δt=1 μs is similar to the 8-thread CPU-based JEMRIS and GPU-based JEMRIS. As the number of voxels increases, the relative overhead of GPU parallelization decreases. With $N_{v}=4\cdot 10^{4}$, GAMS reaches parity with GPU-based JEMRIS, achieving a 5-fold speedup over 8-thread CPU-based JEMRIS. For $N_{v}\geq 10^{6}$, this improvement reaches up to 15-fold over the CPU implementation while maintaining performance equal to GPU-based JEMRIS. On the available hardware, GPU-based JEMRIS failed to complete the simulation for $N_{v}>10^{7}$, whereas GAMS successfully manages it up to $N_{v}=10^{8}$.

## 4. Results and Discussion

After the validation phase, we apply GAMS in three MRI experiments aimed at (i) the quantitative estimation of parameters $T_{1}$ and $T_{2}$, (ii) the handling of distortions in the static magnetic field, and (iii) the imaging of complex anatomical structures. The same scan parameters as in the validation tests are used, namely a static magnetic field amplitude of 1.5 T, a maximum gradient amplitude of 32 $\mathrm{mT}\;m^{-1}$, and a maximum gradient slew rate of 130 $T\;m^{-1}s^{-1}$.

### 4.1. Quantitative Estimation of Relaxation Times from Synthetic Images

Here, we apply GAMS in the generation of synthetic images of a calibration phantom with known material properties, under qMRI-type sequences, and in inverse reconstruction problems based on MR relaxation techniques. The final objective is the quantitative estimation of $T_{1}$ and $T_{2}$ from the generated images, using their “true” values as a reference for accuracy evaluation.

**Figure 6:**
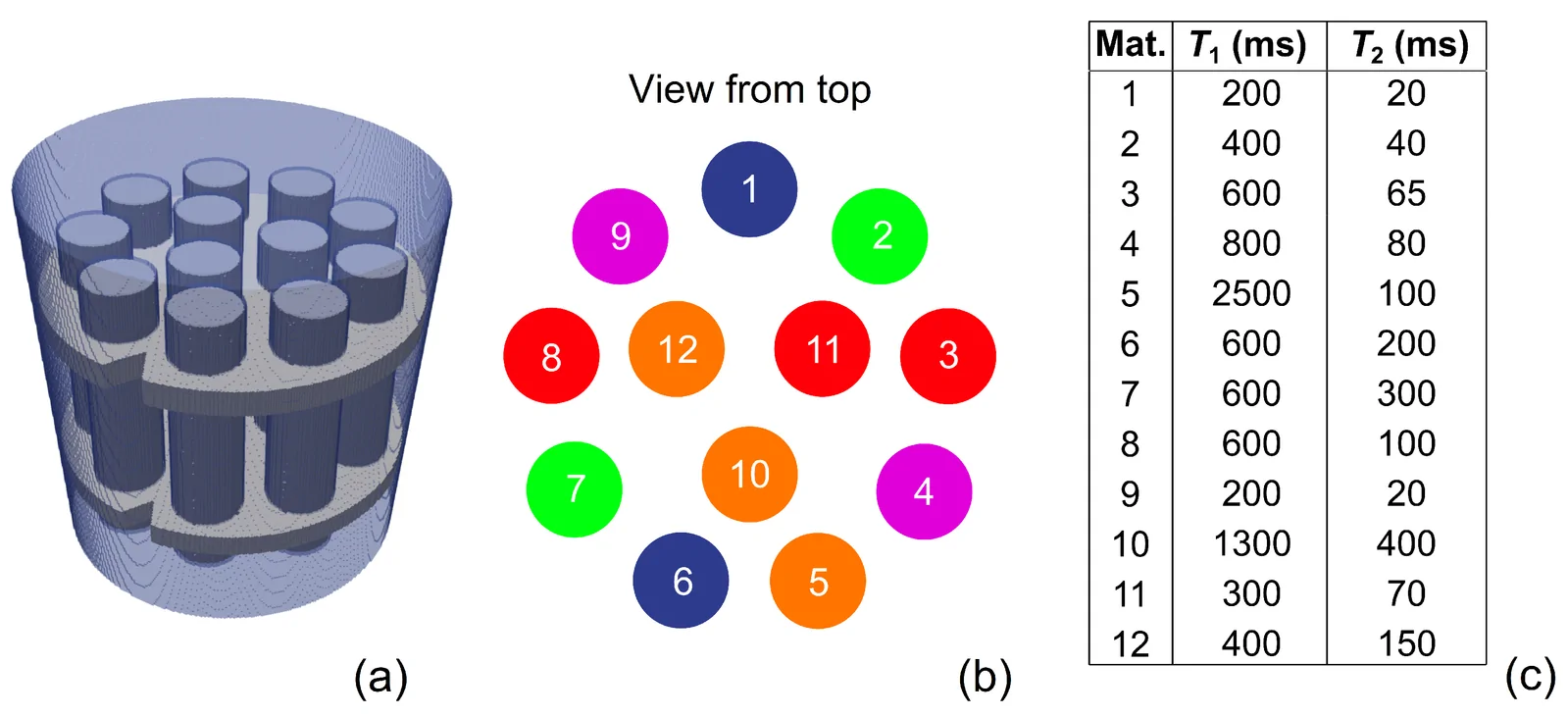
(**a**) Schematic of the calibration phantom used for the quantitative estimation of $T_{1}$ and $T_{2}$. (**b**) Top view of the twelve cylindrical vials positioned within the phantom, with indication of the internal material codes. (**c**) Table of the $T_{1}$ and $T_{2}$ parameters of the materials contained inside the vials.

The phantom consists of a plastic container with the shape of an inverted truncated cone having 129 mm height, 112 mm top diameter and 97 mm bottom diameter. Inside the container, there are twelve cylindrical polypropylene vials, with 18 mm diameter and 90 mm height, held in place by two polyethylene spacers with corresponding holes (Figure 6a); the remaining part is filled with water. Nine vials are arranged on the vertices of a centered regular nonagon with 36 mm circumradius; the other three vials are placed on the vertices of a centered equilateral triangle with 15 mm circumradius (Figure 6b). The physical counterpart is described in [51]. Each vial is filled with a material with values of $T_{1}$ and $T_{2}$ listed in the table in Figure 6c, and PD = 1 p.u. For the polypropylene vials, we assume $T_{1}$ = 5 s, $T_{2}$ = 0.5 s, and PD = 0.001 p.u., while for the polyethylene spacers $T_{1}$ = 5 s, $T_{2}$ = 1 s, and PD = 0.001 p.u. The digital phantom is discretized in voxels of 0.4375×0.4375×0.5 ${\mathrm{mm}}^{3}$.

In the following, we apply two inverse reconstruction methods for the mapping of $T_{1}$ and $T_{2}$, starting from two corresponding sets of synthetic images generated with Inversion Recovery (IR) and Spin Echo (SE) pulse sequences, respectively. Specific analysis techniques of MR relaxometry [26] are then applied to the two sets of images, one for each relaxation time, to estimate the $T_{1}$ and $T_{2}$ parameters of the materials contained in the vials. The realism and ability of GAMS to produce the correct contrast are evaluated qualitatively by comparing the generated images against real MR images.

#### 4.1.1. Quantitative Estimation of $T_{2}$

We simulate a series of Spin Echo (SE) sequences (illustrated in Figure S3 of the Supplementary Materials) to acquire images of a 2 mm thick slice of the phantom’s cross-section, thanks to the inclusion of slice-selective gradients. Images are generated at specific echo times ($T_{E}$) of 10, 20, 50, 120, 320, and 500 ms, with a k-space matrix size of 128×128. To minimize the influence of longitudinal recovery, the repetition time ($T_{R}$) is extended to 5 s. The signal gray intensity, *S*, encoded in the images decays as a function of $T_{E}$ according to the following equation (Section 8.2, [26]):

(6) $$S\left(T_{E}\right)=S\left(0\right)e^{-T_{E}/T_{2}}.$$

Consequently, the $T_{2}$ value is extracted by fitting this exponential equation to the acquired data using the procedures detailed below. Twelve regions of interest (ROIs) are selected, manually masking the areas corresponding to the materials within the twelve vials, and using the same mask for all the images; the generic *j*-th ROI, which should be characterized by a unique value of spin-spin relaxation time $T_{2}^{j}$, contains $N_{j}$~100 pixels. For the estimation of $T_{2}^{j}$, two fitting methodologies are implemented.

With methodology #1, we apply a pixel-level fitting. Specifically, for the generic *k*-th pixel belonging to the *j*-th ROI, we perform a fit with the local gray level intensity function $S^{j,k}\left(T_{E}\right)=S^{j,k}\left(0\right)e^{-T_{E}/T_{2}^{j,k}}$. We use a “Trust-Region” algorithm to solve the non-linear least-squares problem, with the pixel intensity at t=0, $S^{j,k}\left(0\right)$, and $T_{2}^{j,k}$ as the local fitting parameters. In the *j*-th ROI, the reconstructed value of $T_{2}^{j}$ is estimated as ${\bar{T}}_{2}^{j}=\frac{1}{N_{j}}\sum_{k=1}^{N_{j}}T_{2}^{j,k}$.

With methodology #2, we apply an ROI-level fitting. Specifically, at each fixed value of $T_{E}$, the average signal gray level intensity in the generic *j*-th ROI, ${\bar{S}}^{j}\left(T_{E}\right)$, is evaluated and fitted with the average intensity function ${\bar{S}}^{j}\left(T_{E}\right)={\bar{S}}^{j}\left(0\right)e^{-T_{E}/{\bar{T}}_{2}^{j}}$, using ${\bar{S}}^{j}\left(0\right)$ and ${\bar{T}}_{2}^{j}$ as the average fitting parameters. The results obtained by applying the two methodologies are summarized in Table 1, where they are compared to the known “true” values of $T_{2}$; a maximum relative error in the order of 7% is found, and for most of the materials the relative error is lower than 3%.

**Figure 7:**
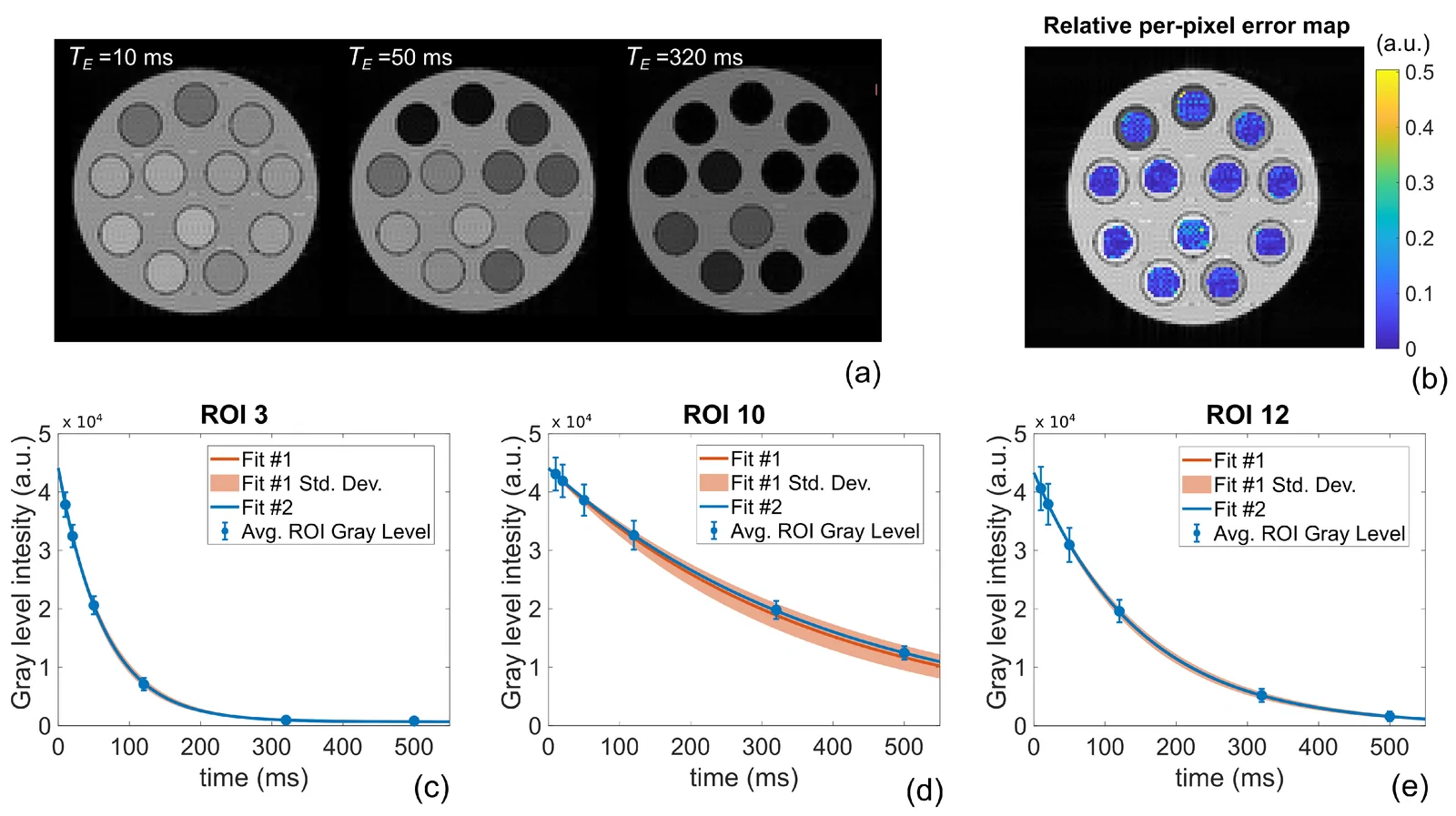
(**a**) DICOM images of the calibration phantom acquired with the SE sequence at spin echo times $T_{E}$ equal to 10, 50 and 320 ms (grayscale images with 65,536 levels are produced). (**b**) Map of the relative per-pixel error of the $T_{2}$ values, estimated with fitting methodology #1. Results of the $T_{2}$ reconstruction for the ROIs No. (**c**) 3, (**d**) 10 and (**e**) 12. The graphs compare the two fitting methodologies, including the average signal gray level intensities (blue dots) as a reference.

As a more detailed investigation, Figure 7a shows the DICOM images generated at spin echo time $T_{E}$ equal to 10, 50 and 320 ms, where it can be seen that the vial internal regions characterized by lower values of $T_{2}$ tend to become dark earlier, due to the shorter relaxation processes. To have a local indication of the accuracy of the quantitative estimation procedure, Figure 7b reports the map of the relative error with respect to the “true” $T_{2}$ value, evaluated at pixel level (fitting methodology #1) in each of the manually selected ROIs. The ROIs No. 1 and 9, corresponding to the lowest value of $T_{2}$, as well as ROI No. 10, corresponding to the highest value of $T_{2}$, are characterized by an overall larger relative per-pixel error (>35% in proximity to the vial wall), associated with a poorer fit and a less accurate estimation of the average value. For ROI No. 10, the latter can be strongly improved by applying fitting methodology #2 (see Table 1).

The average signal gray level intensity and standard deviation extracted from ROIs No. 3, 10 and 12 are depicted separately in Figure 7c–e, as a function of the successive echo times. For the generic *j*-th ROI, we also report the fitting curve obtained by applying methodology #1 and the estimated value ${\bar{T}}_{2}^{j}$, together with the area corresponding to the 95% confidence interval. As a comparison, we add the exponential curve determined by interpolating the average signal intensities (methodology #2). The signals acquired with a short $T_{E}$ have a wider gray level intensity distribution in each ROI than those acquired with a long $T_{E}$.

#### 4.1.2. Quantitative Estimation of $T_{1}$

We simulate a series of Inversion Recovery (IR) sequences (schematic in Figure S4 of the Supplementary Materials), acquiring images of a 2 mm thick slice of the phantom’s cross-section, thanks to the inclusion of slice-selective gradients, at $T_{I}$ equal to 35, 75, 100, 125, 150, 250, 1000, 1500, 2000 and 3000 ms, with a k-space matrix size of 128×128. The same segmentation and ROIs used for the $T_{2}$ value estimation are used to group the image pixels after applying the following transformation to the data extracted from the images

(7) $$\tilde{S}\left(T_{I}+T_{E}\right)=\left\{\begin{array}{cc} -S(T_{I}+T_{E}) & \;0\leq T_{I}<{\tilde{T}}_{I}\\ S(T_{I}+T_{E}) & \;T_{I}\geq {\tilde{T}}_{I} \end{array}\right.,$$

where ${\tilde{T}}_{I}$ is defined such that $\tilde{S}\left({\tilde{T}}_{I}+T_{E}\right)=0$. As clearly illustrated by the example reported in Figure S5 in the Supplementary Materials, this data processing enables us to obtain a signal obeying a simple exponential law, which can be fitted by

(8) $$\tilde{S}\left(T_{I}+T_{E}\right)=A+Be^{-T_{I}/T_{1}},$$

where the fitting parameters are, in addition to $T_{1}$, *A* and *B*, which depend on S(0), $T_{2}$ and $T_{E}$ (Section 8.4, [26]).

Both the pixel-level (#1) and ROI-level (#2) fitting methodologies described in Section 4.1.1 are used. The results obtained for the different ROIs are listed in Table 2, where a maximum relative error in the order of 3% is found, corresponding to one of the ROIs (No. 9) associated with the material having the lowest value of $T_{1}$.

**Figure 8:**
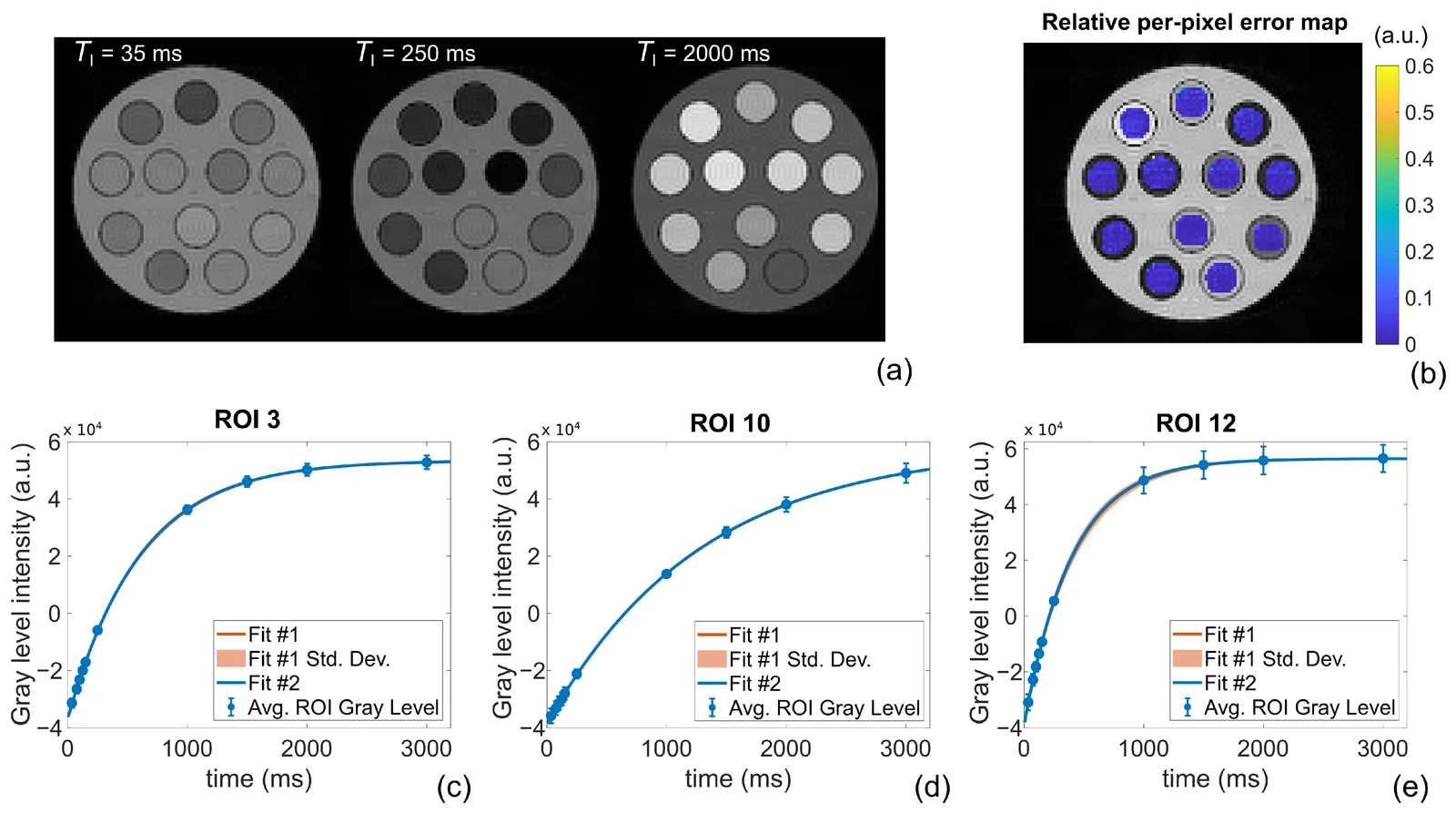
(**a**) DICOM images of the calibration phantom acquired with the IR sequence at inversion times $T_{I}$ equal to 35, 250 and 2000 ms (grayscale images with 65,536 levels are produced). (**b**) Map of the relative per-pixel error of the $T_{1}$ values, estimated with fitting methodology #1. Results of the $T_{1}$ reconstruction for the ROIs numbered (**c**) 3, (**d**) 10 and (**e**) 12. The graphs compare the two fitting methodologies, including the average signal gray level intensities (blue dots) as a reference. The gray level intensity reported in the graphs varies between −65,535 and 65,535; these are obtained for each ROI by applying the data transformation described in Equation (7) and in Figure S5 of the Supplementary Materials.

A subset of the generated DICOM images is reported in Figure 8a, for $T_{I}$ equal to 35, 250 and 2000 ms. We can observe that the image contrast qualitatively reproduces the effects driven by the spin-lattice relaxation time. Ideal signal suppression can be obtained when the inversion time is equal to $\ln\left(2\right)T_{1}$; for example, when $T_{I}$ = 250 ms, the ROI No. 11, corresponding to a value of $T_{1}$ of 300 ms, is very dark, indicating, as expected, good signal suppression, as $\ln\left(2\right)T_{1}$ and $T_{I}$ are very close.

The map of the relative error with respect to the “true” $T_{1}$ value evaluated at pixel level (methodology #1) is shown in Figure 8b; the error is overall lower than in the $T_{2}$ reconstruction experiment, being <1% except for some pixels close to the vial wall. The average signal gray level intensity and standard deviation extracted from the ROIs No. 3, 10 and 12 are depicted separately in Figure 8c–e, as a function of the successive inversion times, confirming the better performance in the quantitative estimation of $T_{1}$. As in the $T_{2}$ reconstruction experiment, in each ROI, the width of the gray level intensity distribution is proportional to the signal amplitude, showing a large error bar when the ROI is bright, which reduces progressively as the ROI becomes dark. The behavior as a function of $T_{I}$ is non-monotonic, since the ROI brightness reduces when $T_{I}$ approaches the critical value, then increases again after that.

#### 4.1.3. Physics-Based Origin of Image Artifacts and Reconstruction Errors

In both the relaxometry experiments for the reconstruction of $T_{1}$ and $T_{2}$, the “true” values are contained in the confidence region of the parameter fit. However, the estimate of $T_{2}$ shows an overall higher variance compared to the $T_{1}$ one, especially for the ROIs characterized by the extreme $T_{2}$ values, which are not optimally sampled by the selected spin echo times. Ideally, one could use more images acquired at longer/shorter times to better reconstruct the high/low values of $T_{2}$; however, this approach would significantly lengthen the measurement process, which is not always feasible, especially in clinical diagnostics. Shifting the acquisition time $T_{E}$ to improve the estimation of the higher $T_{2}$ values would negatively affect the reconstruction of the lower ones and vice versa. As a proof, we perform an experiment similar to the one in Section 4.1.1, maintaining the total number of acquired images, and reading the signal at $T_{E}$ equal to 20, 50, 120, 320, 500 and 1200 ms. With fitting methodology #1, we observe an increase in the relative error up to 15% for the ROIs containing materials with the lowest value of $T_{2}$, i.e., No. 1 and 9. On the contrary, for the ROIs No. 7 and 10, corresponding to the highest values of $T_{2}$, the error reduces to 0.3% and 0.8%, respectively.

The discrepancies found in the $T_{1}$ and $T_{2}$ parameter fit can be attributed to Gibbs artifacts causing the non-uniform gray level in the ROIs, appearing as a striped pattern in both the frequency and phase encode directions. Gibbs ringing is a common artifact in MRI imaging; here, it depends on the presence of sharp interfaces between the material inside vials, the vial walls, and the surrounding water (more generally on a sharp transition in material properties), as well as on the finite sampling in the Fourier domain used to reconstruct the image [52]. The presence of Gibbs ringing in the generated images is a consequence of the realism of the numerical model implemented in the MRI image reconstruction pipeline. Moreover, the 3D simulation with the application of slice-selective gradients naturally captures non-ideal slice profile imperfections. Thus, the final reconstructed image intensity, and consequently the reconstructed relaxation parameters, reflects the true composite signal due to the non-uniform excitation, providing a physically realistic representation of slice-selective signal dynamics.

In real MRI systems, thermal noise can significantly impact the receiver chain, deteriorating $T_{1}$ or $T_{2}$ reconstruction. The data produced with GAMS can be used to analyze hardware noise propagation, i.e., adding zero-mean complex Gaussian noise to the simulated raw k-space data prior to image reconstruction, generating magnitude images with Rician noise distributions at various Signal-to-Noise Ratios (SNRs). As reported in Table S2 of the Supplementary Materials, reducing the SNR introduces significant variance and relative error in the fitted $T_{2}$ values. At lower SNRs (e.g., SNR ≤ 50), the precision of the exponential fit degrades severely across the ROIs and makes the application of methodology #1 more difficult. This behavior accurately reflects a well-known physical limitation in qMRI: at low SNR, the highly attenuated signals at later echo times hit the positive Rician noise floor. Because standard non-linear least-squares exponential fitting does not account for this non-zero baseline, the fit becomes heavily biased and unstable.

#### 4.1.4. Comparison with Real MRI Acquisition

**Figure 9:**
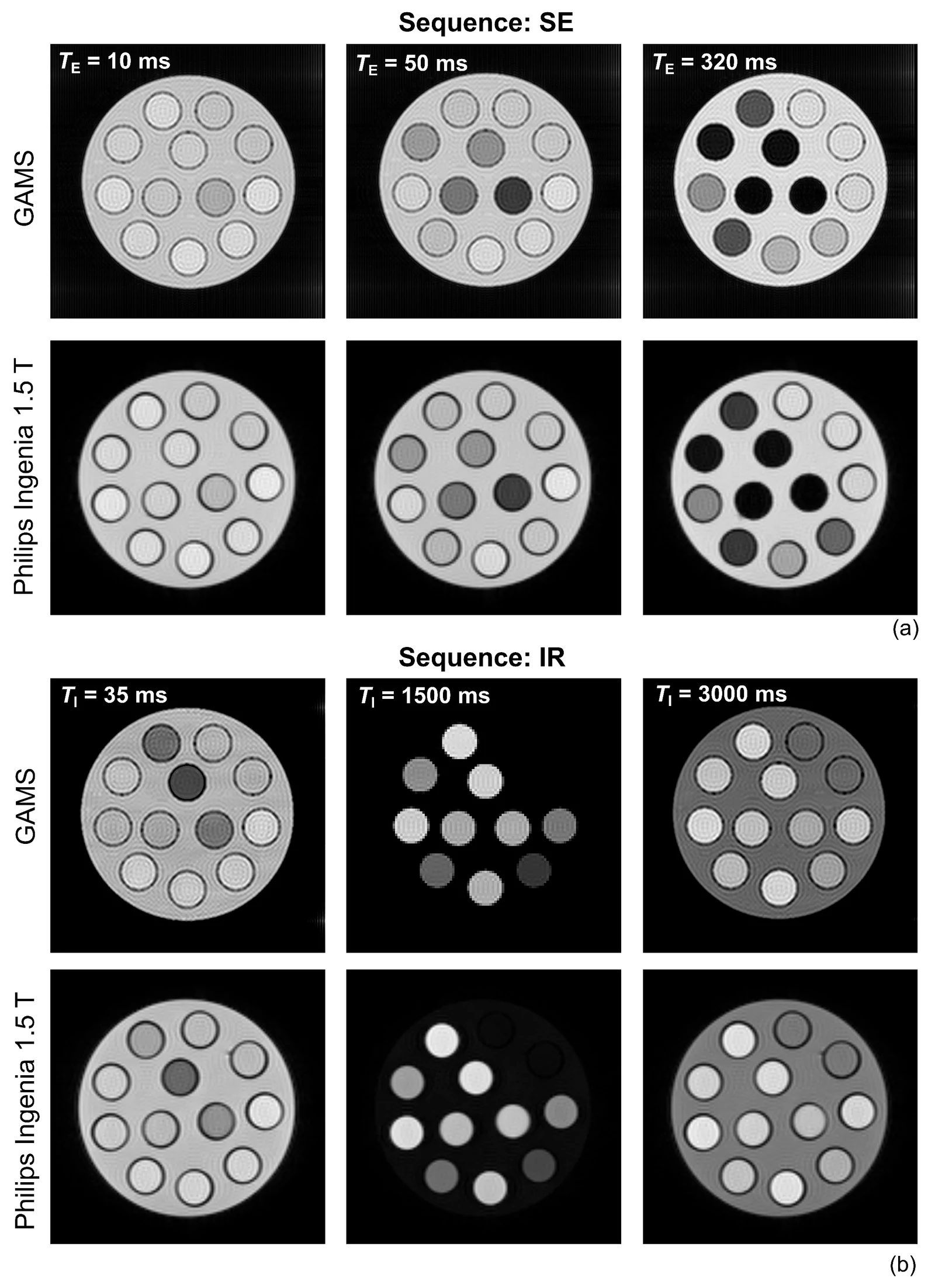
Comparison between the images acquired with a Philips Ingenia 1.5 T scanner using the SE (**a**) and IR (**b**) relaxometry sequences described in Section 4.1.1 and Section 4.1.2 on a calibration phantom used for relaxometry measurements, and the images obtained with GAMS reproducing the same sequences on the digital phantom.

To provide real data, the physical realization of the calibration phantom represented in Figure 6 and described in [51] is imaged with a Philips Ingenia 1.5 T scanner (Philips Medical Systems, Eindhoven, The Netherlands), replicating the experiments described in Section 4.1.1 and Section 4.1.2. The 12 vials are filled with a combination of the $T_{1}$ relaxation ${\mathrm{NiCl}}_{2}$ and $T_{2}$ relaxation ${\mathrm{MnCl}}_{2}$ solutions described by Clarkson et al. [51]. The $T_{1}$ and $T_{2}$ values, extracted by performing the relaxometry analysis described above on the acquired real images, are reported in Table S3 of the Supplementary Materials. The reconstructed values are consistent with the reference ones [51] and are used as input for the simulations. Figure 9 shows the comparison between the real acquired images obtained by applying the two relaxometry sequences and the results of our simulation.Visual comparison reveals a slight misalignment between the physical and simulated phantoms, including a minor rotation of a few degrees and a slight mismatch in the position of the vials. However, the relative contrast of the different solutions is correctly reproduced.

### 4.2. Simulation of Effects of Distortion in the Static Field

**Figure 10:**
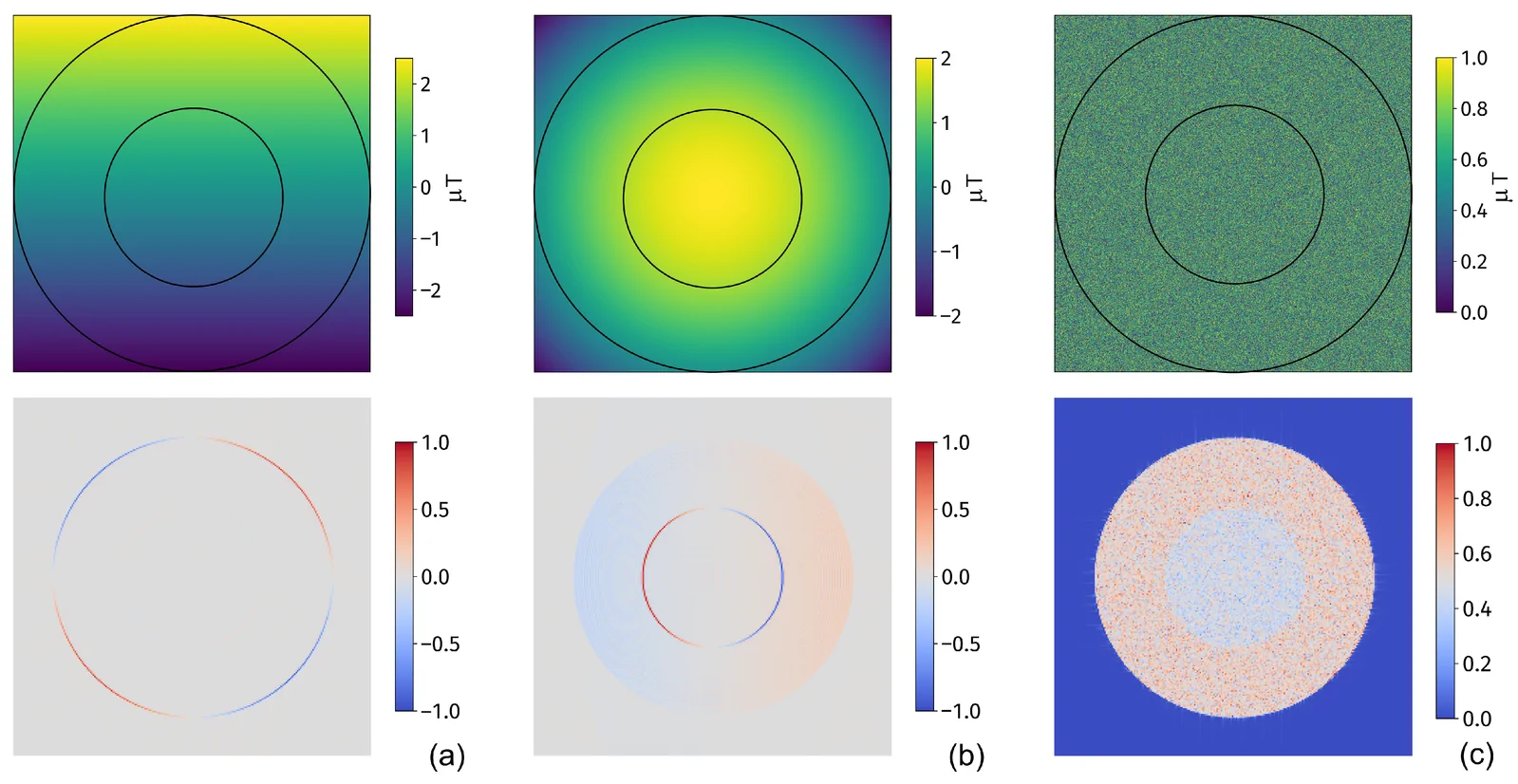
**Top**: maps of the inhomogeneities added to the static field $B_{0}$ corresponding to (**a**) a linear distortion applied along the phase-encoding direction, (**b**) a parabolic distortion with peak at the center of the phantom, and (**c**) white noise. **Bottom**: relative maps of the normalized pixel difference between the images obtained with only $B_{0}$ and with the application of the respective distortion fields.

The flexibility of GAMS in handling distortions in the static field $B_{0}$ is analyzed by imaging a 1 mm thick slice of the high-resolution two-concentric-disk-based phantom of Section 3.2. The images are acquired using the same GRE sequence with a resolution of 256×256 px. Three spatially inhomogeneous fields $\Delta B_{0}=\Delta B_{0}{\hat{z}}_{\mathrm{rot}}$ are added to $B_{0}$, namely a gradient of 50 $\mu T\;m^{-1}$ along the phase-encoding direction (Figure 10a, top), a parabolic distortion centered on the phantom with a maximum variation of 4 μT (Figure 10b, top), and a white noise with an amplitude of 1 μT (Figure 10c, top). At the bottom, we report the maps of the normalized pixel difference between the images obtained with only $B_{0}$ and with the application of the respective distortion fields.

The first applied distortion produces a positive Larmor frequency shift on the phantom top side and a negative shift on the bottom side, with the shift magnitude increasing towards the phantom boundary. This causes a slight leftward displacement of the resulting image in areas with a positive shift and a slight rightward displacement in areas with a negative shift, forming the four-quadrant pattern visible in the difference map at the phantom boundary. A similar effect occurs at the interface between the inner and outer disks, with an opposite sign due to the contrast reversal; because of the very low frequency shift in the inner region, this cannot be appreciated in the current visualization. The second applied distortion generates a positive Larmor frequency shift at the center of the phantom, gradually reducing to zero at the boundary. This creates a leftward shift of the inner disk, noticeable at the inner–outer disk interface. Since the Larmor frequency shift varies along the frequency-encoding direction, a slight gradient in pixel gray levels appears, becoming more pronounced towards the phantom boundary. The third applied distortion is responsible for random Larmor frequency shifts within the phantom, giving rise to a noisy image. The effects described above align well with theoretical predictions. Given an acquisition sequence bandwidth of 62.5 $\mathrm{Hz}\;{\mathrm{px}}^{-1}$, the applied field distortion induces a maximum off-resonance frequency shift of ~100 Hz for the first and second cases, corresponding to a maximum spatial shift of ~1.7 px, which is reflected in the corresponding maps in Figure 10.

### 4.3. Synthetic Imaging of a Realistic Digital Brain Phantom

**Figure 11:**
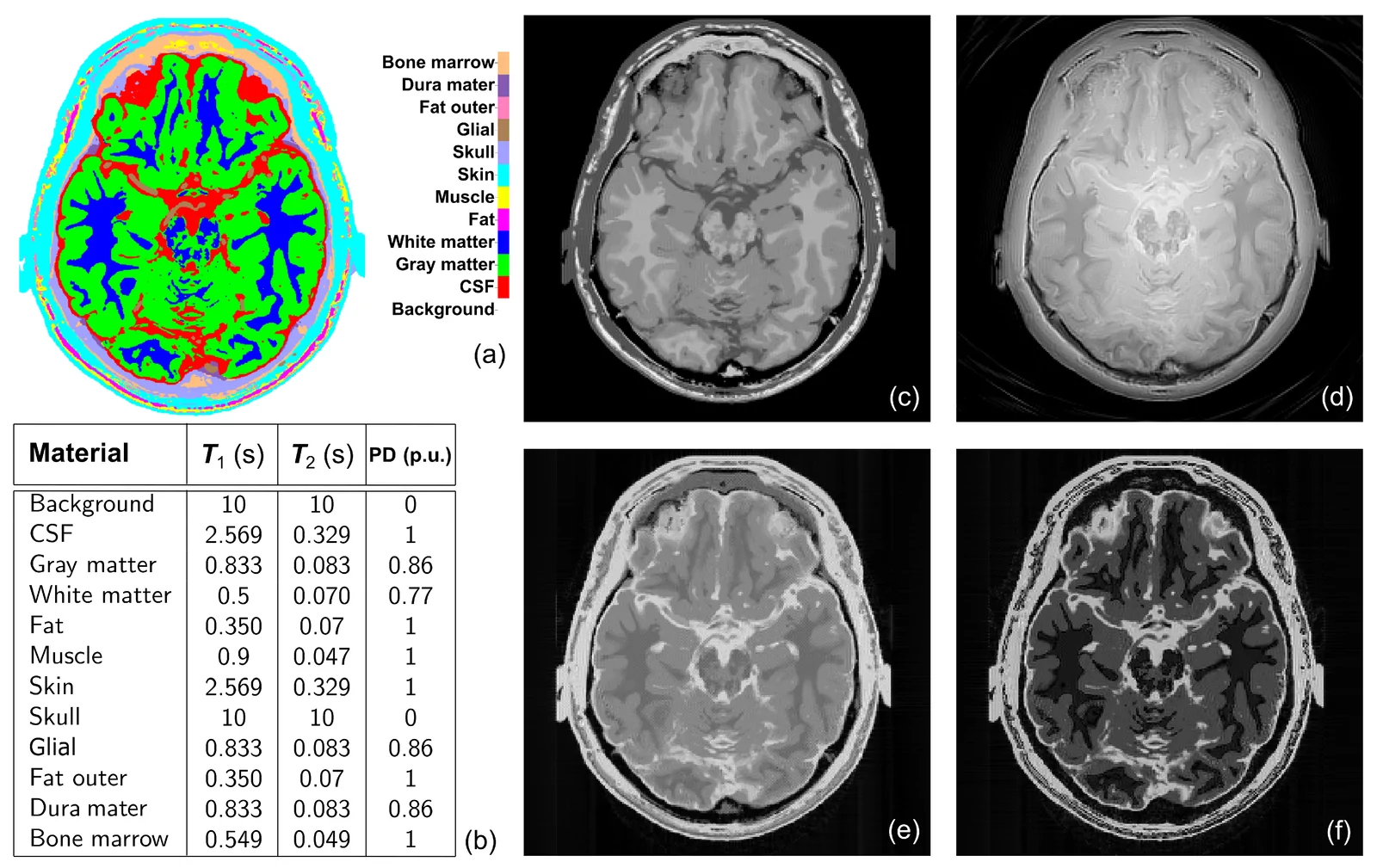
(**a**) Central cross-section of the digital brain phantom, corresponding to the $G_{z}z=0$ plane of the imaged slice, with different tissues shown in false colors. (**b**) Table with the list of tissue properties ($T_{1}$, $T_{2}$ and PD). (**c**) $T_{1}$-weighted image generated with sequence #1 (GRE with $T_{E}$ = 6 ms and $T_{R}$ = 300 ms). (**d**) PD-weighted image generated with sequence #2 (GRE with $T_{E}$ = 8 ms and $T_{R}$ = 5000 ms). (**e**) $T_{2}$-weighted image generated with sequence #3 (SE with $T_{E}$ = 50 ms and $T_{R}$ = 5000 ms). (**f**) White-matter-suppressed image generated with sequence #4 (IR with $T_{E}$ = 7 ms, $T_{R}$ = 4500 ms and $T_{I}$ = 300 ms).

We apply the developed MRI simulator in the generation of synthetic images of complex anatomical structures, using as an input a realistic digital brain phantom with a voxel discretization of 0.5 mm. This corresponds to one of the models (Subject 4) of the segmented human brains gathered at McGill University [21,22] “BrainWeb: 20 Anatomical Models of 20 Normal Brains”. A cross-section of the phantom, corresponding to the imaged slice of 2 mm thickness, is shown in Figure 11a; the tissue parameters provided with the model are listed in the table in Figure 11b.

In order to generate different contrast-weighted images, as a function of the phantom relaxation times, we select the following four MRI pulse sequences:

- Sequence #1: $T_{1}$-weighted GRE with $T_{E}$ = 6 ms and $T_{R}$ = 300 ms;
- Sequence #2: PD-weighted GRE with a 24-arm spiral trajectory, $T_{E}$ = 8 ms and $T_{R}$ = 5000 ms;
- Sequence #3: $T_{2}$-weighted SE with $T_{E}$ = 50 ms and $T_{R}$ = 5000 ms;
- Sequence #4: IR with $T_{E}$ = 7 ms, $T_{R}$ = 4500 ms and $T_{I}$ = 300 ms.

The PyPulseq codes used to generate the above sequences are available in a specific section of the Supplementary Materials. In the $T_{1}$-weighted GRE sequence (#1), a short echo time $T_{E}$ is chosen to minimize the dependency on the transverse relaxation time $T_{2}$, while the short $T_{R}$ allows the magnetization longitudinal component to recover in a way governed principally by the $T_{1}$ relaxation time, reaching a value lower than $M_{0}$. Therefore, after the RF pulse application, the signal intensity mainly depends on the recovered part, enabling the generation of a $T_{1}$-weighted image [46], as depicted in Figure 11c. It is possible to observe that the fat and fat-like tissues, which rapidly realign their longitudinal magnetization with the static magnetic field, have a bright appearance. Conversely, water undergoes a slower realignment of longitudinal magnetization after the RF pulse, leading to reduced transverse magnetization and, consequently, to a lower signal. As a result, cerebrospinal fluid (CSF) and water-rich tissues, like muscles and skin, have a darker appearance. In addition to $T_{1}$, the proton density also influences the signal intensity, $M_{0}$ being proportional to PD, as outlined in Equation (2).

The PD-weighted Spiral GRE sequence (#2) is characterized by a long $T_{R}$ and a short $T_{E}$; thus, the signal intensity dependence on both $T_{1}$ and $T_{2}$ is minimized, enabling the production of a PD-weighted image, strongly correlated with the density of excitable hydrogen atoms in tissues. As shown in Figure 11d, the tissues appear from brighter to darker when moving from higher to lower PD values.

In the $T_{2}$-weighted SE sequence (#3), a long $T_{R}$ is chosen to allow the full recovery of the magnetization, thus reducing the signal intensity dependency on $T_{1}$. Meanwhile, the long $T_{E}$ allows the transverse relaxation processes to develop, enabling the differentiation of the tissues from each other as a function of $T_{2}$, and thus resulting in a $T_{2}$-weighted image [53,54]. In clinical practice, this sequence is indeed used to identify diseased areas where the pathological condition is responsible for an increase in the water content, thus appearing brighter than the surrounding tissues. This behavior is confirmed for the considered brain model slice, as highlighted by the contrast differences in the synthetic image of Figure 11e. The white matter has a short $T_{2}$, and therefore it dephases quickly and appears dark; CSF, which is a watery substance, has a long $T_{2}$ and appears very bright; gray matter, which is characterized by intermediate $T_{2}$ values, appears medium gray.

In the IR sequence (#4), a short $T_{E}$ is chosen to minimize the signal dependence on $T_{2}$ [54]. The selected $T_{I}$ is tuned to suppress the white matter signal and reduce the signal intensity from other tissues characterized by a value of $T_{1}$ in the range 350–800 ms. As expected, the obtained image (Figure 11f) shows the white matter almost black and a great separation between the fatty regions with a short $T_{1}$, which appear dark, and muscle, skin, and CSF that have a long $T_{1}$, and thus appear bright.

For sequences #3 and #4, the signal gray level intensity *S* can be described analytically by Equations (6) and (8), respectively. In Figure S6 of the Supplementary Materials, the images generated with GAMS are compared to an ideal relative gray level map. The analytical map of *S* is calculated for each phantom voxel corresponding to the middle plane of the imaged 2 mm thick slice, and then rescaled to match the image resolution of the simulated MRI sequence. Similar contrast is exhibited, with global SSIM values (calculated excluding the background) of 0.805 for sequence #3 and 0.794 for sequence #4. The relatively low scores can be attributed to Gibbs ringing effects and FFT-based reconstruction artifacts, which typically occur in MRI image acquisition but are not present in the ideal map. Furthermore, the numerically calculated images are affected by the combined contributions of multiple voxels along the z-axis, with possibly different tissue composition. This leads to a blending effect responsible for a diminution in image sharpness, as happened in the frontal lobes where a gradual transition is observable, with a consequent decrease in local SSIM values (see the maps in Figure S6).

Overall, all the obtained DICOM images show in an accurate way the details of the brain structure, correctly reproducing the contrast between tissues, according to their properties and to the applied MRI pulse sequences.

## 5. Conclusions

We present an MRI simulator, GAMS, developed in a CUDA Fortran environment and optimized for GPU-based computation acceleration. The use of GPU parallelization enables us to reduce the computational time by two orders of magnitude in comparison to a single-threaded CPU implementation. The developed pipeline closely follows the process of generating real MRI images, requiring sequence-specific information, to provide a reliable tool able to support the validation of MRI measurement techniques and the testing of scanner performance. Particular care has been taken to ensure compatibility with community-driven standards, guaranteeing the use of Pulseq format for MRI pulse sequence design.

The benchmark of GAMS in comparison to the analytical solution available for a FID problem demonstrates its high level of accuracy, with numerical errors controlled by the single-precision limit. Good agreement has also been found with the well-established MRI simulator JEMRIS, with a very low pixel-by-pixel difference between the synthetic images obtained with the two numerical codes.

GAMS has proven to be a valuable tool also for the quantitative estimation of the relaxation properties of test objects, like calibration phantoms containing very different materials, which can be used for the generation of reference synthetic data to support inter-laboratory comparisons. It can be applied to investigate off-resonance artifacts caused by spatial inhomogeneity of the static magnetic field, and their impact on image quality. While GAMS presently assumes ideal, homogeneous RF fields, explicit $B_{1}^{+}$ transmit profiles can be included in the effective field formulation, and complex $B_{1}^{-}$ receive coil sensitivities can be directly integrated into the final signal acquisition. Moreover, with GAMS it is possible to test and prototype a wide variety of MRI pulse sequences and evaluate their role in DICOM image generation as a function of phantom composition. This has been proven in the case of a high-resolution digital brain phantom, for which images with different contrast and weighting have been synthesized, depending on the adopted sequence parameters and the tissues to be highlighted.

As a future application, GAMS can be adopted for the generation of synthetic images with the aim of expanding medical datasets for artificial intelligence model validation through the implementation of extended virtual clinical trials.

## Figures and Tables

**Table 1:** Comparison between the “true” $T_{2}$ values of the materials contained in the twelve vials of the calibration phantom and the values reconstructed via relaxometry experiment simulation. The fitted values and the relative percentage errors obtained with the two fitting methodologies are reported.

ROI (Mat.)	True $T_{2}$ (ms)	Fit #1 (ms)	Fit #1 (Error)	Fit #2 (ms)	Fit #2 (Error)
1	20	18.61	6.95%	18.57	7.15%
2	40	38.42	3.95%	38.41	3.97%
3	65	64.1	1.38%	63.91	1.67%
4	80	78.29	2.13%	78.24	2.2%
5	100	98.02	1.98%	97.04	2.96%
6	200	197.3	1.35%	199	0.5%
7	300	297.8	0.73%	300.4	0.13%
8	100	97.68	2.32%	97.13	2.87%
9	20	18.9	5.5%	18.91	5.45%
10	400	382.3	4.42%	401.3	0.32%
11	70	68.27	2.47%	68.32	2.4%
12	150	150	0.00%	150.4	0.26%

**Table 2:** Comparison between the “true” $T_{1}$ values of the materials contained in the twelve vials of the calibration phantom and the values reconstructed via relaxometry experiment simulation. The fitted values and the relative percentage errors obtained with the two fitting methodologies are reported.

ROI (Mat.)	True $T_{1}$ (ms)	Fit #1 (ms)	Fit #1 (Error)	Fit #2 (ms)	Fit #2 (Error)
1	200	196.87	1.57%	196.76	1.62%
2	400	396.16	0.96%	393.6	1.6%
3	600	603.22	0.54%	601.57	0.26%
4	800	803.85	0.48%	801.65	0.2%
5	2500	2484.9	0.6%	2479.1	0.84%
6	600	602.51	0.42%	597.26	0.46%
7	600	604.08	0.68%	602.65	0.44%
8	600	597.17	0.47%	594.51	0.92%
9	200	205.83	2.92%	205.07	2.54%
10	1300	1281.7	1.4%	1280.3	1.51%
11	300	302.65	0.88%	301.84	0.61%
12	400	403.72	0.93%	398.34	0.42%

## Data Availability

The raw data supporting the conclusions of this article will be made available by the authors upon request. The analysis scripts and the PyPulseq scripts used to generate the MRI sequences and data are available at https://github.com/Morrighan89/GAMS_public (accessed on 27 September 2026).

## Supplementary Materials

The following supporting information can be downloaded at: https://www.mdpi.com/article/10.3390/jimaging12100477/s1, Figure S1: GRE sequence scheme; Figure S2: Comparison GAMS-JEMRIS low resolution concentric disk; Figure S3: SE sequence scheme; Figure S4: IR sequence scheme; Figure S5: $T_{1}$ signal pre-processing; Figure S6: Comparison of MRI simulation and analytical attenaution maps; Table S1: Material IDs and properties of JEMRIS’ brain phantom; Table S2: $T_{2}$ relaxometry percentage error as function of SNR; Table S3: Material properties extracted from physical phantom. Some of the scripts used in this article are listed at the end.

## Abbreviations

The following abbreviations are used in this manuscript:

ACQ	Data Acquisition Period.
DICOM	Digital Imaging and Communications in Medicine.
FID	Free Induction Decay.
FFT	Fast Fourier Transform.
FOV	Field Of View.
GAMS	GPU-Accelerated MRI Simulator.
GPU	Graphics Processing Unit.
GRE	Gradient Recalled Echo.
IR	Inversion Recovery Spin Echo.
JEMRIS	Jülich Extensible MRI Simulator.
MRI	Magnetic Resonance Imaging.
PD	Proton Density.
PyPulseq	Python Package for MRI Pulse Sequence Design.
qMRI	Quantitative MRI.
RF	Radiofrequency.
ROI	Region Of Interest.
SE	Spin Echo.
$T_{1}$	Longitudinal Relaxation Time.
$T_{2}$	Transverse Relaxation Time.
$T_{E}$	Echo Time.
$T_{I}$	Inversion Time.
$T_{R}$	Repetition Time.

## References

[1] Granziera C., Wuerfel J., Barkhof F., Calabrese M., De Stefano N., Enzinger C., Evangelou N., Filippi M., Geurts J.J.G., Reich D.S. (2021). Quantitative magnetic resonance imaging towards clinical application in multiple sclerosis. Brain.

[2] Otto L.A., van der Pol W.L., Schlaffke L., Wijngaarde C.A., Stam M., Wadman R.I., Cuppen I., van Eijk R.P., Asselman F.L., Bartels B. (2020). Quantitative MRI of skeletal muscle in a cross-sectional cohort of patients with spinal muscular atrophy types 2 and 3. NMR Biomed..

[3] Seiler A., Nöth U., Hok P., Reiländer A., Maiworm M., Baudrexel S., Meuth S., Rosenow F., Steinmetz H., Wagner M. (2021). Multiparametric Quantitative MRI in Neurological Diseases. Front. Neurol..

[4] Shrestha U., Esparza J.P., Satapathy S.K., Vanatta J.M., Abramson Z.R., Tipirneni-Sajja A. (2024). Hepatic steatosis modeling and MRI signal simulations for comparison of single- and dual-R2* models and estimation of fat fraction at 1.5T and 3T. Comput. Biol. Med..

[5] Keenan K.E., Ainslie M., Barker A.J., Boss M.A., Cecil K.M., Charles C., Chenevert T.L., Clarke L., Evelhoch J.L., Finn P. (2018). Quantitative magnetic resonance imaging phantoms: A review and the need for a system phantom. Magn. Reson. Med..

[6] Keenan K.E., Gimbutas Z., Dienstfrey A., Stupic K.F., Boss M.A., Russek S.E., Chenevert T.L., Prasad P.V., Guo J., Reddick W.E. (2021). Multi-site, multi-platform comparison of MRI T1 measurement using the system phantom. PLoS ONE.

[7] Boonsuth R., Battiston M., Grussu F., Samlidou C.M., Calvi A., Samson R.S., Gandini Wheeler-Kingshott C.A.M., Yiannakas M.C. (2023). Feasibility of in vivo multi-parametric quantitative magnetic resonance imaging of the healthy sciatic nerve with a unified signal readout protocol. Sci. Rep..

[8] Thomas D.C., Deichmann R., Nöth U., Langkammer C., Ferreira M., Golbach R., Hattingen E., Wenger K.J. (2024). A fast protocol for multicenter and multiparametric quantitative MRI studies in brain tumor patients using vendor sequences. Neuro-Oncol. Adv..

[9] Marchant J.K., Rosen B.R. (2025). Bridging the resolution gap in alpha therapy dosimetry: A space for quantitative MRI?. Phys. Med. Biol..

[10] Cao Z., Park J., Cho Z.H., Collins C.M. (2015). Numerical evaluation of image homogeneity, signal-to-noise ratio, and specific absorption rate for human brain imaging at 1.5, 3, 7, 10.5, and 14T in an 8-channel transmit/receive array. J. Magn. Reson. Imaging.

[11] Restivo M.C., Ramasawmy R., Bandettini W.P., Herzka D.A., Campbell-Washburn A.E. (2020). Efficient spiral in-out and EPI balanced steady-state free precession cine imaging using a high-performance 0.55T MRI. Magn. Reson. Med..

[12] Vallières M., Laberge S., Diamant A., El Naqa I. (2017). Enhancement of multimodality texture-based prediction models via optimization of PET and MR image acquisition protocols: A proof of concept. Phys. Med. Biol..

[13] Spronk T., Kraff O., Schaefers G., Quick H.H. (2023). Numerical approach to investigate MR imaging artifacts from orthopedic implants at different field strengths according to ASTM F2119. Magn. Reson. Mater. Phys. Biol. Med..

[14] Keskin K., Sanson A.R., Hargreaves B.A., Nayak K.S. (2026). Open-Source Simulator of Imaging Near Metal at Arbitrary Magnetic Field Strengths. Magn. Reson. Med..

[15] Schmidt J., Radunsky D., Scheibe P., Jäger C., Ben-Eliezer N., Trampel R., Weiskopf N. (2025). High-resolution quantitative T2 mapping of the human brain at 7 T using a multi-echo spin-echo sequence and dictionary-based modeling. Imaging Neurosci..

[16] Scalco E., Rizzo G., Mastropietro A. (2023). The quantification of IntraVoxel incoherent motion—MRI maps cannot preserve texture information: An evaluation based on simulated and *in-vivo* images. Comput. Biol. Med..

[17] Lin Q., Chen W., Kang T., Wu J., Chen X., Qu X., Lin L., Wang J., Lin J., Chen Z. (2025). Fast water/fat T2 and PDFF mapping via multiple overlapping-echo detachment acquisition and deep learning reconstruction. Phys. Med. Biol..

[18] Fieremans E., Lee H.H. (2018). Physical and numerical phantoms for the validation of brain microstructural MRI: A cookbook. NeuroImage.

[19] Keenan K.E., Wilmes L.J., Aliu S.O., Newitt D.C., Jones E.F., Boss M.A., Stupic K.F., Russek S.E., Hylton N.M. (2016). Design of a breast phantom for quantitative MRI. J. Magn. Reson. Imaging.

[20] Stupic K.F., Ainslie M., Boss M.A., Charles C., Dienstfrey A.M., Evelhoch J.L., Finn P., Gimbutas Z., Gunter J.L., Hill D.L.G. (2021). A standard system phantom for magnetic resonance imaging. Magn. Reson. Med..

[21] Aubert-Broche B., Evans A.C., Collins L. (2006). A new improved version of the realistic digital brain phantom. NeuroImage.

[22] Aubert-Broche B., Griffin M., Pike G., Evans A., Collins D. (2006). Twenty New Digital Brain Phantoms for Creation of Validation Image Data Bases. IEEE Trans. Med. Imaging.

[23] Sainz Martinez C., Bach Cuadra M., Jorge J. (2023). BigBrain-MR: A new digital phantom with anatomically-realistic magnetic resonance properties at 100-µm resolution for magnetic resonance methods development. NeuroImage.

[24] Lu M., Xiao X., Song H., Liu G., Lu H., Kikkawa T. (2021). Accurate construction of 3-D numerical breast models with anatomical information through MRI scans. Comput. Biol. Med..

[25] Oria M., Ferrero R., Andreis C., Vicentini M., van Engen R., Roozemond C., Lamberti P., Remogna S., Manzin A. (2025). Image-based reconstruction of anthropomorphic breast phantoms for synthetic mammogram generation. Comput. Biol. Med..

[26] Brown R.W., Cheng Y.C.N., Haacke E.M., Thompson M.R., Venkatesan R. (2014). Magnetic Resonance Imaging: Physical Principles and Sequence Design.

[27] Petersson J.S., Christoffersson J.O., Golman K. (1993). MRI simulation using the k-space formalism. Magn. Reson. Imaging.

[28] Morris G.A., Chilvers P.B. (1994). General Analytical Solutions of the Bloch Equations. J. Magn. Reson. A.

[29] Benoit-Cattin H., Collewet G., Belaroussi B., Saint-Jalmes H., Odet C. (2005). The SIMRI project: A versatile and interactive MRI simulator. J. Magn. Reson..

[30] Stöcker T., Vahedipour K., Pflugfelder D., Shah N.J. (2010). High-performance computing MRI simulations. Magn. Reson. Med..

[31] Chen Z., Chen Z. (2021). Computed inverse MRI (CIMRI) for intrinsic brain magnetic susceptibility mapping. Comput. Biol. Med..

[32] Li J.R., Calhoun D., Poupon C., Le Bihan D. (2013). Numerical simulation of diffusion MRI signals using an adaptive time-stepping method. Phys. Med. Biol..

[33] Graham M.S., Drobnjak I., Zhang H. (2016). Realistic simulation of artefacts in diffusion MRI for validating post-processing correction techniques. NeuroImage.

[34] Kose R., Setoi A., Kose K. (2018). A Fast GPU-optimized 3D MRI Simulator for Arbitrary k-space Sampling. Magn. Reson. Med. Sci..

[35] Xanthis C.G., Aletras A.H. (2019). coreMRI: A high-performance, publicly available MR simulation platform on the cloud. PLoS ONE.

[36] Castillo-Passi C., Coronado R., Varela-Mattatall G., Alberola-López C., Botnar R., Irarrazaval P. (2023). KomaMRI.jl: An open-source framework for general MRI simulations with GPU acceleration. Magn. Reson. Med..

[37] Duncan-Gelder P., O’Keeffe D., Bones P., Marsh S. (2024). PhoenixMR: A GPU-based MRI simulation framework with runtime-dynamic code execution. Med. Phys..

[38] Nurdinova A., Ruschke S., Gestrich M., Stelter J., Karampinos D.C. (2025). Gpu-accelerated JEMRIS for extensive MRI simulations. Magn. Reson. Mater. Phys. Biol. Med..

[39] Huang H., Yang Q., Wang J., Zhang P., Cai S., Cai C. (2023). High-efficient Bloch simulation of magnetic resonance imaging sequences based on deep learning. Phys. Med. Biol..

[40] Manzin A., Ferrero R., Vicentini M. (2021). From Micromagnetic to In Silico Modeling of Magnetic Nanodisks for Hyperthermia Applications. Adv. Theory Simul..

[41] Ferrero R., Manzin A. (2021). Adaptive geometric integration applied to a 3D micromagnetic solver. J. Magn. Magn. Mater..

[42] Ferrero R., Vicentini M., Manzin A. (2024). Influence of size, volume concentration and aggregation state on magnetic nanoparticle hyperthermia properties versus excitation conditions. Nanoscale Adv..

[43] Cashmore M.T., McCann A.J., Wastling S.J., McGrath C., Thornton J., Hall M.G. (2021). Clinical quantitative MRI and the need for metrology. Br. J. Radiol..

[44] Layton K.J., Kroboth S., Jia F., Littin S., Yu H., Leupold J., Nielsen J.F., Stöcker T., Zaitsev M. (2017). Pulseq: A rapid and hardware-independent pulse sequence prototyping framework. Magn. Reson. Med..

[45] Ravi K.S., Geethanath S., Vaughan J.T. (2019). PyPulseq: A Python Package for MRI Pulse Sequence Design. J. Open Source Softw..

[46] Bernstein M.A., King K.F., Zhou Z.J. (2004). Handbook of MRI Pulse Sequences.

[47] Paschal C.B., Morris H.D. (2004). K-space in the clinic. J. Magn. Reson. Imaging.

[48] Fessler J., Sutton B. (2003). Nonuniform fast Fourier transforms using min-max interpolation. IEEE Trans. Signal Process..

[49] Beatty P., Nishimura D., Pauly J. (2005). Rapid gridding reconstruction with a minimal oversampling ratio. IEEE Trans. Med. Imaging.

[50] Wang Z., Bovik A., Sheikh H., Simoncelli E. (2004). Image quality assessment: From error visibility to structural similarity. IEEE Trans. Image Process..

[51] Clarkson C., Hill S., Gezer T.D., Ün I., Rai S., Abukashabeh A., Entwisle J., Cashmore M., Cooke E.A., McDowell A.R. (2025). A standard SI traceable phantom suitable for qMRI: Design, manufacture and characterisation. Metrologia.

[52] McRobbie D.W., Moore E.A., Graves M.J., Prince M.R. (2017). MRI from Picture to Proton.

[53] Bitar R., Leung G., Perng R., Tadros S., Moody A.R., Sarrazin J., McGregor C., Christakis M., Symons S., Nelson A. (2006). MR Pulse Sequences: What Every Radiologist Wants to Know but Is Afraid to Ask. Radiographics.

[54] Chen Y., Almarzouqi S.J., Morgan M.L., Lee A.G., Schmidt-Erfurth U., Kohnen T. (2018). T2-Weighted Image. Encyclopedia of Ophthalmology.

