# Supplementary material for: A GPU-Accelerated MRI Simulator for Synthetic Image Generation
Source: J Imaging. 2026 Oct 1;12(10):477. doi: 10.3390/jimaging12100477 (PMC13706494; doi:10.3390/jimaging12100477)
Supplement: Supplementary file 1 [file jimaging-12-00477-s001.zip › jimaging-4459300-supplementary.pdf]

# Supplementary Material: A GPU-accelerated MRI simulator for synthetic image generation

Riccardo Ferrero[1,*], Marta Vicentini[1], Elizabeth Cooke[2], Cormac McGrath[3], Aaron McCann[3], Amy McDowell[4], Nick Zafeiropoulos[4], Paul Tofts[5], Matt G. Hall[2], Alessandra Manzin[1]

[1]Istituto Nazionale di Ricerca Metrologica (INRiM), Strada delle Cacce 91, 10135 Torino, Italy
[2]National Physical Laboratory (NPL), Teddington, United Kingdom
[3]Belfast Health and Social Care Trust, Belfast, United Kingdom
[4]University College London, London, United Kingdom
[5]Brighton and Sussex Medical School, Brighton, United Kingdom

## Figures

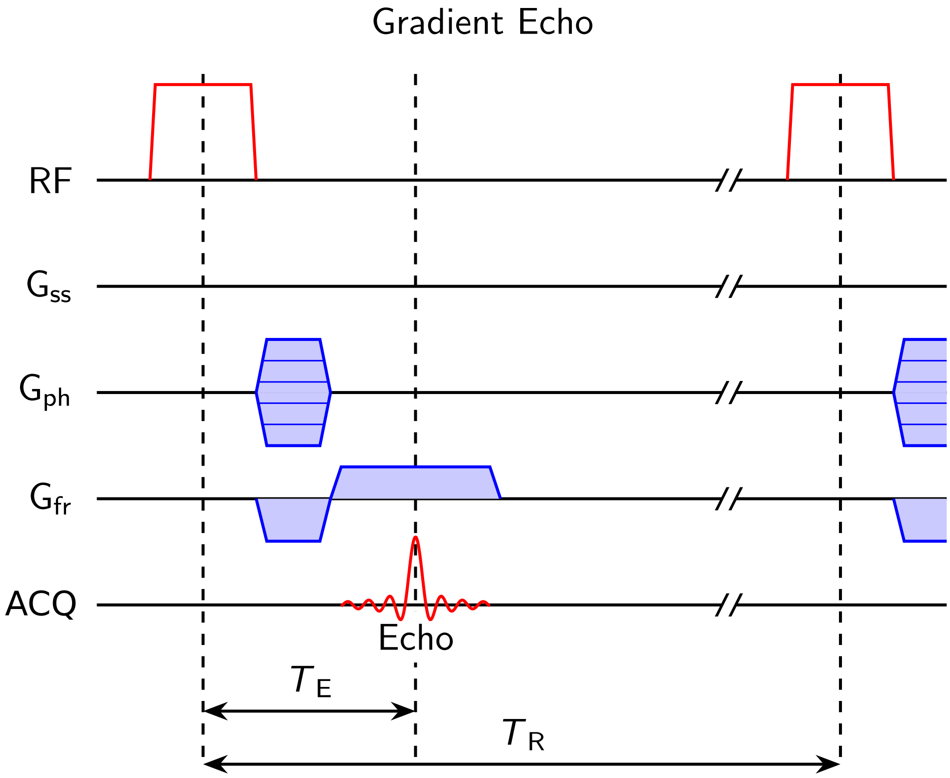


Figure S1: Schematic of the Gradient Echo (GRE) sequence applied in Subsection 3.2. A constant amplitude RF pulse is applied along $z$-axis, without any slice-selection gradient ($G_{ss}$), since the phantom corresponds to a slice excited in all its thickness. The amplitude of the phase-encoding gradient ($G_{ph}$), which is applied along $y$-axis, varies after each repetition and is represented as a stack of trapezoidal functions. The frequency-encoding or read-out gradient ($G_{fr}$) is applied along $x$-axis. ACQ indicates the data acquisition period. The time interval representation is reported for illustrative purposes.

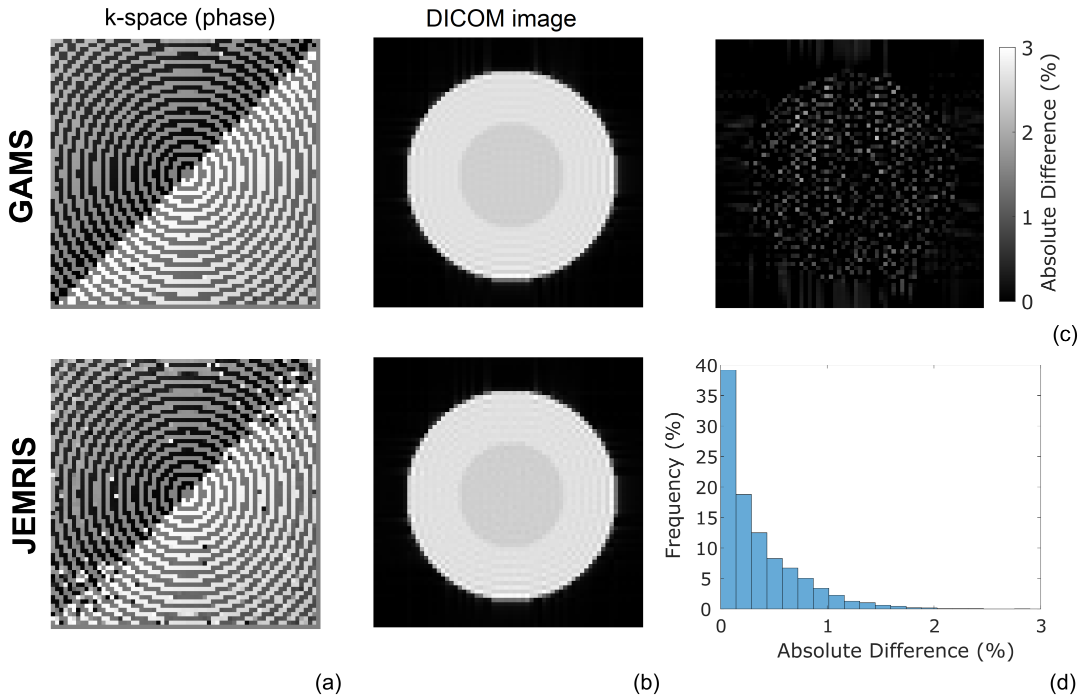


Figure S2: Comparison between the simulation results obtained with GAMS and JEMRIS, by applying a GRE sequence for the 64×64 px imaging of the two concentric disk-based phantom with 1 mm voxel size, used for GAMS validation in Section 3.2. (a) Maps of the k-space phase. (b) DICOM images. (c) Pixel-by-pixel map of the absolute difference in grey level intensity between the obtained images. (d) Frequency plot of the absolute difference at pixel level.

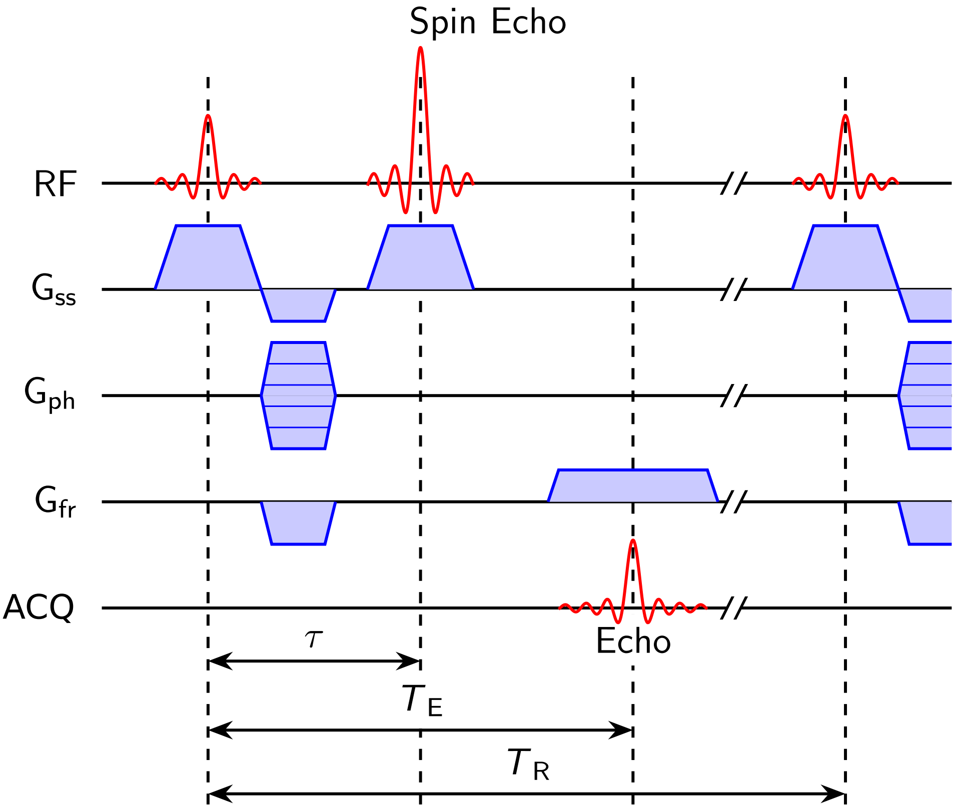


Figure S3: Schematic of the Spin Echo (SE) sequence applied in Subsection 4.1 for the $T_2$ reconstruction experiment. A sync-shaped RF pulse is applied, with a slice-selection gradient ($G_{ss}$) along $z$-axis to excite only a section of the phantom. This flips the magnetization of 90° in the $xy$-plane. The phase-encoding ($G_{ph}$) and frequency-encoding ($G_{fr}$) gradients are applied along the $y$- and $x$-axes, respectively. The amplitude of the phase-encoding gradient varies after each repetition and is represented as a stack of trapezoidal functions. A secondary 180° rotating RF sync pulse is applied, to refocus the magnetization, then the read-out gradient is applied in the frequency-encoding direction along the $x$-axis. ACQ indicates the data acquisition period. The time interval representation is reported for illustrative purposes.

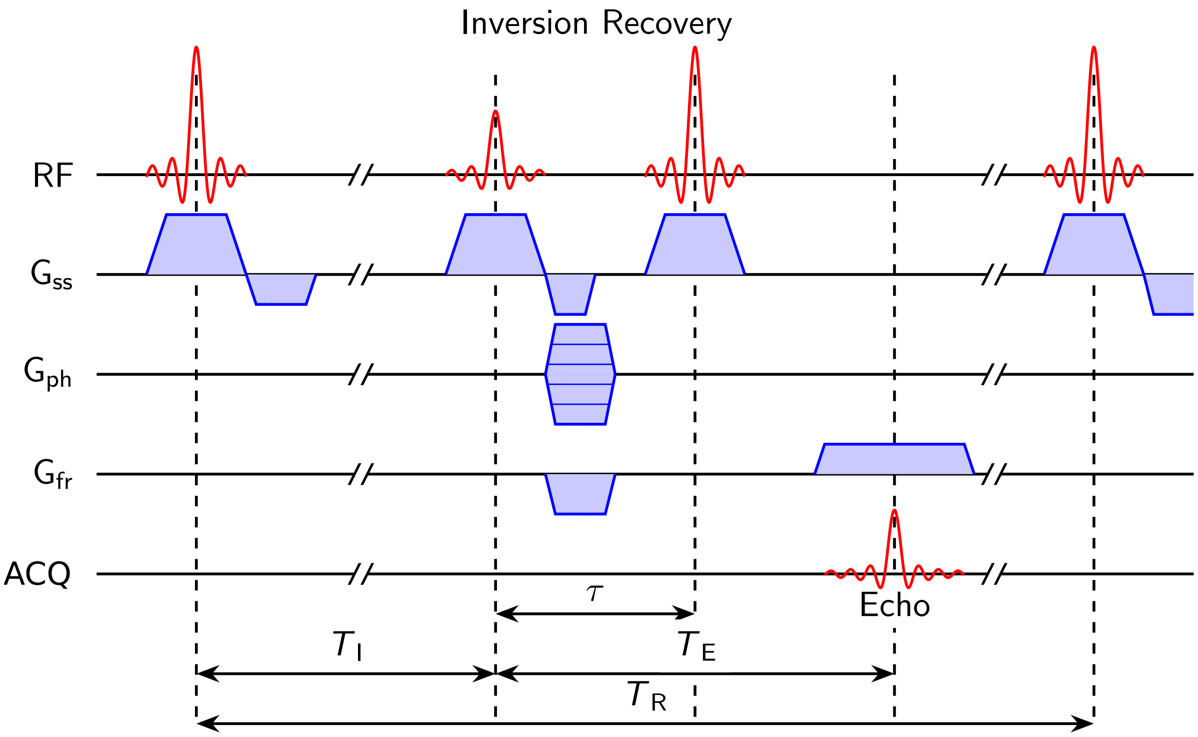


Figure S4: Schematic of the Inversion Recovery Spin Echo (IRSE) sequence applied in Subsection 4.1 for the $T_1$ reconstruction experiment. The magnetization is flipped of 180° and let to relax towards the equilibrium for a certain time called Inversion Time ($T_{\mathrm{I}}$). After $T_{\mathrm{I}}$, an SE sequence is applied, with the magnetization flipped in the $xy$-plane, resulting in a signal strongly dependent on $T_{\mathrm{I}}$ and $T_1$. ACQ indicates the data acquisition period. The time interval representation is reported for illustrative purposes.

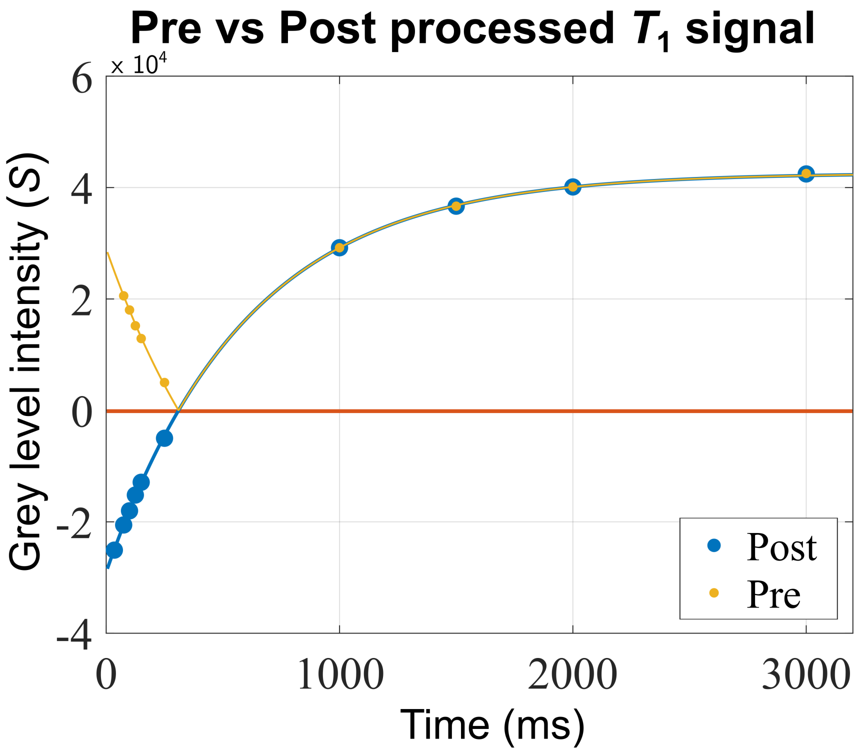


Figure S5: Pre- and post-processed grey level intensity $S$ at pixel level (example reported as support for the $T_1$ reconstruction experiment reported in Subsection 4.1). $S$ is transformed to obey a simple exponential law shown in Equation (8). The yellow dots represent the signal intensity data extracted from the images acquired at different $T_\mathrm{I}$ values. The blue dots represent the series of values obtained after applying the transformation described in Equation (7). A candidate value for $\tilde{T}_\mathrm{I}$ is initially selected equal to the minima of the original function. After the transformation, we check that the first derivative of the new function is strictly monotonically decreasing. The derivative is approximated by the angular coefficient of the line passing between two subsequent values of $S$. The estimate is then corrected accordingly with an iterative procedure.

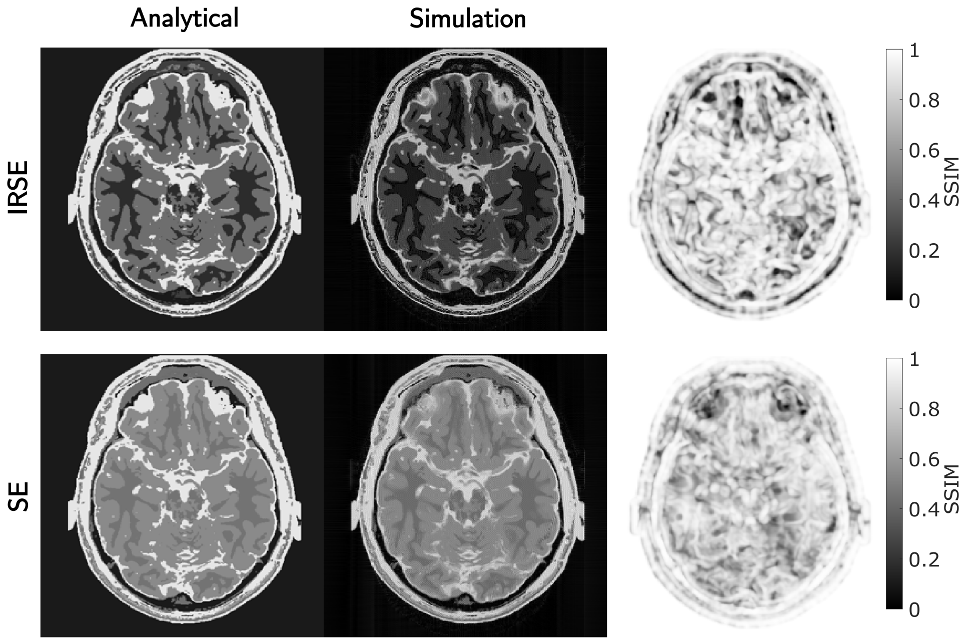


Figure S6: Comparison between the analytical attenuation maps of the tissues for the IRSE (first row) and SE (second row) sequences and the MRI image obtained from simulations. The last column shows the SSIM map between each pair of images.

# Tables

Table S1: Material codes with relative properties ($T_1$, $T_2$ and PD) of the brain model available in the JEMRIS simulation framework, and used in Subsection 3.2 for GAMS validation.

| ROI (Mat.) | $T_1$ (s) | $T_2$ (s) | PD (p.u.) |
|---|---|---|---|
| 0 | 20 | 20 | 0 |
| 1 | 0.5 | 0.07 | 0.77 |
| 2 | 0.833 | 0.083 | 0.86 |
| 3 | 2.569 | 0.329 | 1 |
| 4 | 2.569 | 0.329 | 1 |
| 5 | 0.9 | 0.047 | 1 |
| 6 | 0.35 | 0.07 | 1 |

Table S2: Percentage error of methodology #2 of $T_2$ fitting for different levels of Signal to Noise Ratio (SNR).

| ROI (Mat.) | Zero Noise | SNR 70 (21 dB) | SNR 50 (16 dB) | SNR 22 (14 dB) | SNR 16 (8.4 dB) | SNR 7 (1.2 dB) | SNR 5 (-2 dB) |
|---|---|---|---|---|---|---|---|
| 1 | 7.15 % | 14.99 % | 14.71 % | 22.49 % | 14.4 % | 27.81 % | 45.4 % |
| 2 | 3.97 % | 7.79 % | 12.87 % | 24.66 % | 20.39 % | 35.46 % | 51.0 % |
| 3 | 1.67 % | 7.66 % | 6.9 % | 21.31 % | 18.2 % | 24.05 % | 37.41 % |
| 4 | 2.2 % | 5.95 % | 10.16 % | 18.0 % | 22.71 % | 47.02 % | 20.3 % |
| 5 | 2.96 % | 9.19 % | 10.346 % | 22.31 % | 33.09 % | 53.53 % | 31.17 % |
| 6 | 0.5 % | 4.41 % | 1.64 % | 12.3 % | 23.06 % | 44.68 % | 37.69 % |
| 7 | 0.13 % | 2.14 % | 3.87 % | 2.34 % | 11.93 % | 35.1 % | 46.59 % |
| 8 | 2.87 % | 5.32 % | 6.93 % | 17.88 % | 27.7 % | 29.29 % | 42.46 % |
| 9 | 0.45 % | 14.81 % | 16.68 % | 27.94 % | 37.07 % | 28.66 % | 41.27 % |
| 10 | 0.33 % | 1.7 % | 1.25 % | 4.35 % | 15.37 % | 22.66 % | 7.63 % |
| 11 | 2.4 % | 8.11 % | 8.14 % | 19.37 % | 26.37 % | 29.23 % | 61.46 % |
| 12 | 0.27 % | 3.82 % | 4.06 % | 22.16 % | 22.09 % | 41.17 % | 50.66 % |

Table S3: Material codes with relative properties ($T_1$, $T_2$ and PD) measured from the physical calibration phantom used in Subsection 4.1.4 for the comparison with real MRI data.

| ROI (Mat.) | $T_1$ (s) | $T_2$ (s) | PD (p.u.) |
|---|---|---|---|
| 1 | 2.8 | 1 | 1 |
| 2 | 1 | 1 | 1 |
| 3 | 0.64 | 0.64 | 1 |
| 4 | 0.45 | 0.40 | 1 |
| 5 | 0.226 | 0.185 | 1 |
| 6 | 0.11 | 0.094 | 1 |
| 7 | 2.8 | 1 | 1 |
| 8 | 1.5 | 0.3 | 1 |
| 9 | 1.2 | 0.187 | 1 |
| 10 | 0.8 | 0.111 | 1 |
| 11 | 0.58 | 0.063 | 1 |
| 12 | 0.32 | 0.031 | 1 |

## Scripts

List of scripts, written following Pulseq format, for the generation of the MRI pulse sequences used in the simulations.
These scripts are also available here `https://github.com/Morrighan89/GAMS_public`

### GRE sequence (template used for JEMRIS-GAMS comparison in Subsection 3.2 and for the generation of $T_1$-weighted image in Subsection 4.3)

```
from math import pi
import numpy as np
from matplotlib import pyplot as plt
from pypulseq.Sequence.sequence import Sequence
from pypulseq.calc_duration import calc_duration
from pypulseq.make_adc import make_adc
from pypulseq.make_delay import make_delay
from pypulseq.make_sinc_pulse import make_sinc_pulse
from pypulseq.make_trap_pulse import make_trapezoid
from pypulseq.opts import Opts
"""
USER INPUTS
These parameters are typically on the user interface of the scanner computer console
"""
nsa = 1  # Number of averages
n_slices = 1  # Number of slices
Nx = 256
Ny = 256
fovx = 220.e-3  # mm
fovy = 220.e-3
slice_thickness = 2.e-3  # s
slice_gap = 0.e-3  # s
rf_flip = 60  # degrees
rf_offset = 0
apodization=2
print('User inputs setup')
""" SYSTEM LIMITS
Set the hardware limits and initialize sequence object
"""
system = Opts(max_grad=32, grad_unit='mT/m', max_slew=130, slew_unit='T/m/s',
              rf_ringdown_time=10e-6, grad_raster_time=10.e-6,
              rf_dead_time=100e-6)
seq = Sequence(system)
""" TIME CONSTANTS """
TE = np.array([8.e-3])
TR = 500e-3
""" RF
Provide flip angle in deg, first line converts to radians
The function gives the RF sync, the slice selection gradient gz and the rephasing gradient gzr
"""
flip_ang = round(rf_flip * pi / 180, 3)
rf, gz, gzr = make_sinc_pulse(flip_angle=flip_ang, system=system, duration=1.5e-3, slice_thickness=
     slice_thickness, apodization=apodization,  return_gz=True, time_bw_product=4)
""" READOUT
Readout gradients and related events
"""
delta_kx = 1 / fovx
gx = make_trapezoid(channel='x', flat_area=Nx * delta_kx, flat_time=5.12e-3, system=system)
adc = make_adc(num_samples=Nx, duration=gx.flat_time, delay=gx.rise_time, system=system)
""" PREPHASE AND REPHASE
"""
delta_ky = 1 / fovy
phase_areas = (np.arange(Ny) - Ny / 2) * delta_ky
gx_pre = make_trapezoid(channel='x', area=-gx.area / 2, duration=3.5e-3, system=system)
gz_reph = make_trapezoid(channel='z', area=-gz.area / 2, duration=3.5e-3, system=system)
""" DELAYS
Echo time (TE) and repetition time (TR). Here, TE is broken down into `delay1` and `delay2`.
"""
delay_TE = TE - calc_duration(gx_pre) - gz.fall_time - gz.flat_time / 2 - calc_duration(
    gx) / 2
delay_TR = TR - calc_duration(gz) - calc_duration(gx_pre) - calc_duration(
    gx) - delay_TE
assert np.all(delay_TE >= 0)
#assert np.all(delay_TR >= calc_duration(gx_spoil, gz_spoil))
""" CONSTRUCT SEQUENCE
"""
rf_phase = 0
rf_inc = 0
freq_offset = gz.amplitude * slice_gap
rf.freq_offset = freq_offset
seq.add_block(rf, gz)
seq.add_block(make_delay(delay_TR[0]))
for i in range(Ny):
```

```
    for j in range(len(TE)):
        rf.phase_offset = rf_phase / 180 * np.pi
        adc.phase_offset = rf_phase / 180 * np.pi
        seq.add_block(rf, gz)
        gy_pre = make_trapezoid(channel='y', area=phase_areas[i], duration=calc_duration(gx_pre), system=
            system)
        seq.add_block(gx_pre, gy_pre, gz_reph)
        seq.add_block(make_delay(delay_TE[j]))
        seq.add_block(gx, adc)
        gy_pre.amplitude = -gy_pre.amplitude
        seq.add_block(make_delay(delay_TR[j]))
ok, error_report = seq.check_timing()  # Check whether the timing of the sequence is correct
if ok:
    print('Timing check passed successfully')
else:
    print('Timing check failed. Error listing follows:')
    [print(e) for e in error_report]
""" PLOTTING TIMNG DIAGRAM """
seq.plot(time_range=(delay_TR[0], delay_TR[0]+0.025))
""" GENERATING `.SEQ` FILE
Uncomment the code in the cell below to generate a `.seq` file and download locally.
"""
# Prepare the sequence output for the scanner
seq.set_definition('FOV', [fovx, fovy, slice_thickness])
seq.set_definition('Name', 'GRE')
seq.set_definition('Nx,Ny', [Nx,Ny])
seq.set_definition('flip', [rf_flip])
seq.set_definition('TR,TE', [TR,TE])
seq.set_definition('apodization', apodization)
""" GENERATING `.SEQ` FILE
"""
seq.write(f'GRE_{rf_flip}TE{int(TE*1000):03d}TR{int(TR*1000):04d}_test.seq')
```

## Glover Spiral GRE sequence (template used for the generation of PD-weighted image in Subsection 4.3)

```
import math
import numpy as np
import matplotlib.pyplot as plt
import pypulseq as pp
from pypulseq.make_arbitrary_grad import make_arbitrary_grad
from sigpy.mri.rf.trajgrad import spiral_arch
G_max = 32           # Max Gradient in mT/m
S_max = 130          # Max Slew Rate in T/m/s (equivalent to mT/m/ms for SigPy)
# 1. Define system limits (PyPulseq 1.3.1 syntax)
system = pp.Opts(
    max_grad=G_max, grad_unit='mT/m',
    max_slew=S_max, slew_unit='T/m/s',
    rf_ringdown_time=20e-6,
    rf_dead_time=100e-6,
    adc_dead_time=20e-6
)
# Initialize the sequence
seq = pp.Sequence(system=system)
# 2. Sequence parameters
fov = 220e-3               # Field of view (256 mm)
Nx = 256       # Base resolution
alpha = 15                 # Flip angle in degrees
slice_thickness = 5.e-3    # 5 mm
TE = 8.e-3                 # Echo time
TR = 5000e-3                 # Repetition time
N_interleaves =  24        # Number of spiral interleaves (multi-shot)
#T_readout = 20e-3           # Readout duration per interleave
res = fov / Nx
# 3. Create RF pulse and slice selection gradients
rf, gz, _ = pp.make_sinc_pulse(
    flip_angle=alpha * math.pi / 180,
    duration=3e-3,
    slice_thickness=slice_thickness,
    apodization=0.5,
    time_bw_product=4,
    system=system,
    return_gz=True
)
# Slice rephasing gradient
gz_reph = pp.make_trapezoid(channel='z', area=-gz.area/2, duration=1.5e-3, system=system)
# From Nyquist theorem the k-space must reach kmax=1/(2* delta_x) to be able to resolve spatial features
     with reolution delta_x = FOV/Nx => kmax = Nx/(2*FOV)
kmax = Nx/(2*fov)
gamma = system.gamma  # ~42.576 MHz/T
#--- 2. Calculate Glover Trajectory (via SigPy) ---
# TRICK: To create an interleaved trajectory, a single arm only needs
# to cover an "effective FOV" equal to (Total FOV / Number of Arms).
```

```python
effective_fov = fov / N_interleaves
dt = system.grad_raster_time
# SigPy calculates the safe transition automatically.
# It returns the gradient waveform as a complex array (gx + i*gy) in mT/m.
g_complex, k_out, t_out, s_out = spiral_arch(
    fov=effective_fov,
    res=res,
    gts=dt,
    gslew=S_max*0.91,
    gamp=G_max*0.92
)
# Extract real (X) and imaginary (Y) parts
gx_mT = g_complex[:,0]
gy_mT = g_complex[:,1]
# Convert mT/m to Hz/m (the internal unit PyPulseq expects for custom arrays)
# 1 mT = 1e-3 T. system.gamma is approximately 42.58e6 Hz/T for protons.
gx_base = gx_mT * 1e-3 * system.gamma
gy_base = gy_mT * 1e-3 * system.gamma
# Calculate the duration in milliseconds
readout_ms = len(gx_base) * dt * 1000
print(f"The Glover optimized readout time is: {readout_ms:.2f} ms")
#strip the fake last 0
gx_base=gx_base[:-1]
gy_base=gy_base[:-1]
# --- NEW: PHYSICS-DRIVEN ADC CALCULATION ---
# 1. Find the maximum gradient amplitude in the spiral (usually at the outer edge)
g_max = np.max(np.sqrt(gx_base**2 + gy_base**2))
# 2. Calculate the Nyquist limit for dwell time (PyPulseq gradients are already in Hz/m)
dt_nyquist = 1 / (g_max * fov)
dt_adc_ideal = dt_nyquist
# 3. Round to the standard Pulseq ADC raster time (100 ns)
adc_raster = 100e-9
dt_adc = round(dt_adc_ideal / adc_raster) * adc_raster
# 4. Calculate the final number of ADC points required
N_pts_adc = int(round(readout_ms/1000 / dt_adc))
# 5. Build the Sequence Loop
for i in range(N_interleaves):
    angle = i * (2 * math.pi / N_interleaves)

    gx_rot = gx_base * math.cos(angle) - gy_base * math.sin(angle)
    gy_rot = gx_base * math.sin(angle) + gy_base * math.cos(angle)

    # --- Ramp-Down Calculation ---
    # 1. Get the very last point of the rotated waveforms
    # 2. PREPEND a true zero to the start for a smooth PyPulseq liftoff
    gx_rot = np.concatenate(([0], gx_rot))
    gy_rot = np.concatenate(([0], gy_rot))

    # 3. CALCULATE the ramp down from the true final velocity
    gx_last = gx_rot[-1]
    gy_last = gy_rot[-1]

    g_mag_last = np.sqrt(gx_last**2 + gy_last**2)
    s_max_Hz = S_max * system.gamma

    ramp_time = g_mag_last / s_max_Hz
    n_ramp_pts = int(np.ceil(ramp_time / dt)) + 1

    ramp_x = np.linspace(gx_last, 0, n_ramp_pts)[1:]
    ramp_y = np.linspace(gy_last, 0, n_ramp_pts)[1:]

    gx_safe = np.concatenate((gx_rot, ramp_x))
    gy_safe = np.concatenate((gy_rot, ramp_y))
    gx = pp.make_arbitrary_grad.make_arbitrary_grad(channel='x', waveform=gx_safe, system=system)
    gy = pp.make_arbitrary_grad.make_arbitrary_grad(channel='y', waveform=gy_safe, system=system)

    # Create ADC event matched to the spiral readout, using our new N_pts_adc
    adc = pp.make_adc(num_samples=N_pts_adc, dwell=dt_adc, system=system)

    # Calculate delays (using calc_duration as discussed)
    rf_dur = pp.calc_duration(rf)
    gz_reph_dur = pp.calc_duration(gz_reph)
    gx_duration=pp.calc_duration(gx)
    delay_TE = TE - (rf_dur / 2) - gz_reph_dur
    seq_time_so_far = rf_dur + gz_reph_dur + delay_TE + gx_duration
    delay_TR = TR - seq_time_so_far

    # Append blocks
    seq.add_block(rf, gz)
    seq.add_block(gz_reph)
    if delay_TE > 0:
        seq.add_block(pp.make_delay(delay_TE))

    seq.add_block(gx, gy, adc)

    if delay_TR > 0:
        seq.add_block(pp.make_delay(delay_TR))
```

```
# Check timing and write to .seq file
print(seq.test_report())
seq.plot()
fig, ax = plt.subplots(1, 1,figsize=(7.5,5), layout="constrained")
ktraj_adc, ktraj, t_excitation, t_refocusing, _ = seq.calculate_kspace()
 # Plot the entire k-space trajectory
ax.plot(ktraj[0], ktraj[1], 'b')  # 2D plot
  # Enforce aspect ratio for the correct trajectory display
ax.plot(ktraj_adc[0], ktraj_adc[1], 'r.')
fig.show()
seq.set_definition('FOV', [fov, fov, slice_thickness])
seq.set_definition('Name', 'spiral_gre')
seq.set_definition('Nx,NInterleaves', [Nx, N_interleaves])
seq.set_definition('flip', alpha )
seq.set_definition('TR,TE', [TR, TE])
seq.set_definition('SliceThickness', slice_thickness)
seq.write(f'spiral_greTE{int(TE*1000):03d}TR{int(TR*1000):04d}.seq')
print("Spiral GRE sequence successfully generated and saved to 'spiral_gre.seq'.")
```

## IRSE sequence (template used for quantitative estimate of $T_1$ in Subsection 4.1)

```
from math import pi
import numpy as np
import math
from matplotlib import pyplot as plt
from pypulseq.Sequence.sequence import Sequence
from pypulseq.calc_duration import calc_duration
from pypulseq.make_adc import make_adc
from pypulseq.make_delay import make_delay
from pypulseq.make_sinc_pulse import make_sinc_pulse
from pypulseq.make_trap_pulse import make_trapezoid
from pypulseq.opts import Opts
""" USER INPUTS
These parameters are typically on the user interface of the scanner computer console
"""
nsa = 1  # Number of averages
n_slices = 1  # Number of slices
Nx = 256
Ny = 256
fovx = 220.e-3  # mm
fovy = 220.e-3
slice_thickness = 2.e-3  # s
slice_gap = 0.e-3  # s
rf_flip = 90  # degrees
rf_offset = 0
apodization=1
print('User inputs setup')
""" SYSTEM LIMITS
Set the hardware limits and initialize sequence object
"""
system = Opts(max_grad=32, grad_unit='mT/m', max_slew=130, slew_unit='T/m/s',
              rf_ringdown_time=10e-6, grad_raster_time=10.e-6,
              rf_dead_time=100e-6)
seq = Sequence(system)
""" TIME CONSTANTS """
TE = 7.1e-3  # s
TR = 4.5  # s
TI= 300.e-3
readout_time = 2.5e-3
pre_time = 8e-4  # s
""" RF """
flip90 = round(rf_flip * pi / 180, 3) #round to three decimal digits
flip180 = 180 * pi / 180
rf90, gz90, _ = make_sinc_pulse(flip_angle=flip90, system=system, duration=1.99e-3,
slice_thickness=slice_thickness, apodization=apodization,
return_gz=True,time_bw_product=2, use = 'excitation')
rf180, gz180, _ = make_sinc_pulse(flip_angle=flip180, system=system,
return_gz=True, duration=1.89e-3,  slice_thickness=slice_thickness,
apodization=apodization, time_bw_product=2, use = 'refocusing')
rf_inv,gz_inv,_ = make_sinc_pulse(flip_angle=flip180, system=system,
return_gz=True, duration=2.51e-3,  slice_thickness=slice_thickness,
apodization=apodization,time_bw_product=2, use = 'inversion')
""" READOUT
Readout gradients and related events
"""
delta_kx = 1 / fovx
delta_ky = 1 / fovy
k_width_x = Nx * delta_kx
k_width_y = Ny * delta_ky
gx = make_trapezoid(channel='x', system=system, flat_area=k_width_x,
                    flat_time=readout_time)
adc = make_adc(num_samples=Nx, duration=gx.flat_time, delay=gx.rise_time)
print(gx)
""" PREPHASE AND REPHASE """
```

```
phase_areas = (np.arange(Ny) - (Ny / 2)) * delta_ky
gz_reph = make_trapezoid(channel='z', system=system, area=-gz90.area / 2,
                          duration=readout_time / 2)
gx_pre = make_trapezoid(channel='x', system=system, flat_area=k_width_x / 2,
                         flat_time=readout_time / 2)
gy_pre = make_trapezoid(channel='y', system=system, area=phase_areas[-1],
                         duration=readout_time / 2)
""" DELAYS
Echo time (TE) and repetition time (TR). Here, TE is broken down into `delay1` and `delay2`.
"""
delay_TI = TI - calc_duration(rf_inv) / 2 - calc_duration(rf90) / 2
delay_TE1=TE/2 - calc_duration(rf90) / 2 -calc_duration(gx_pre) - calc_duration(rf180) / 2
delay_TE2=TE/2 - calc_duration(rf180) / 2 - calc_duration(gx)/2

delay_TR = TR -TI - TE - calc_duration(gx)/2
delay_TR = make_delay(delay_TR)
delay_TE1 = make_delay(delay_TE1)
delay_TE2 = make_delay(delay_TE2)
delay_TI = make_delay(delay_TI)
print(f'delay_TI: {delay_TI}')
print(f'delay_TE: {delay_TE1}')
print(f'delay_TE: {delay_TE2}')
print(f'delay_TR: {delay_TR}')
""" CONSTRUCT SEQUENCE
Construct sequence for one phase encode and multiple slices
"""
# Prepare RF offsets. This is required for multi-slice acquisition
delta_z = n_slices * slice_gap
z = np.linspace((-delta_z / 2), (delta_z / 2), n_slices) + rf_offset
#print(phase_areas)
for k in range(nsa):  # Averages
  for j in range(n_slices):  # Slices
    # Apply RF offsets
    freq_offset = gz90.amplitude * z[j]
    rf90.freq_offset = freq_offset
    freq_offset = gz180.amplitude * z[j]
    rf180.freq_offset = freq_offset
    for i in range(Ny):  # Phase encodes
        seq.add_block(rf_inv,gz_inv)
        seq.add_block(delay_TI)
        seq.add_block(rf90, gz90)
        gy_pre = make_trapezoid(channel='y', system=system, area=phase_areas[i], duration=readout_time / 2)
        seq.add_block(gx_pre, gy_pre, gz_reph)
        seq.add_block(delay_TE1)
        seq.add_block(rf180, gz180)
        seq.add_block(delay_TE2)
        seq.add_block(gx, adc)
        seq.add_block(delay_TR)
""" PLOTTING TIMING DIAGRAM """
ok, error_report = seq.check_timing()  # Check whether the timing of the sequence is correct
if ok:
    print('Timing check passed successfully')
else:
    print('Timing check failed. Error listing follows:')
    [print(e) for e in error_report]
seq.plot(time_range=(TR-0.1, 2*TR))
""" GENERATING `.SEQ` FILE
"""
# Prepare the sequence output for the scanner
seq.set_definition('FOV', [fovx, fovy, slice_thickness])
seq.set_definition('Name', 'TSE')
seq.set_definition('Nx,Ny', [Nx,Ny])
seq.set_definition('flip', [rf_flip])
seq.set_definition('TR,TE,TI', [TR,TE,TI])
seq.set_definition('SliceThickness', slice_thickness)
seq.set_definition('apodization', apodization)
TEs=int(TE*1000)
TRs=int(TR*1000)
TIs=int(TI*1000)
seq.write(f'IRSE_{rf_flip}TE{TEs:03d}TR{TRs:04d}TI{TIs:03d}.seq')
```

## SE sequence (template used for quantitative estimate of $T_2$ in Sub-section 4.1 and for the generation of $T_2$-weighted image in Sub-section 4.3)

```
from math import pi
import numpy as np
import math
from matplotlib import pyplot as plt
from pypulseq.Sequence.sequence import Sequence
from pypulseq.calc_duration import calc_duration
from pypulseq.make_adc import make_adc
from pypulseq.make_delay import make_delay
from pypulseq.make_sinc_pulse import make_sinc_pulse
```

```
from pypulseq.make_trap_pulse import make_trapezoid
from pypulseq.opts import Opts
""" USER INPUTS
These parameters are typically on the user interface of the scanner computer console
"""
nsa = 1  # Number of averages
n_slices = 1  # Number of slices
Nx = 256
Ny = 256
fovx = 220.e-3  # mm
fovy = 220.e-3 # mm
slice_thickness = 2.e-3  # s
slice_gap = 0.e-3  # s
rf_flip = 90  # degrees
rf_offset = 0
apodization=2
print('User inputs setup')
""" SYSTEM LIMITS
Set the hardware limits and initialize sequence object
"""
system = Opts(max_grad=32, grad_unit='mT/m', max_slew=130, slew_unit='T/m/s',
              rf_ringdown_time=10e-6, grad_raster_time=10.e-6,
              rf_dead_time=100e-6)
seq = Sequence(system)
""" TIME CONSTANTS """
TE = 50.e-3  # s
TR = 5  # s
tau = TE / 2  # s
readout_time = 4.5e-3
pre_time = 8e-4  # s
""" RF """
flip90 = round(rf_flip * pi / 180, 3) #round to three decimal digits
flip180 = 180 * pi / 180
rf90, gz90, _ = make_sinc_pulse(flip_angle=flip90, system=system, duration=2.51e-3,
                                slice_thickness=slice_thickness, apodization=apodization,
                                return_gz=True,time_bw_product=2)
rf180, gz180, _ = make_sinc_pulse(flip_angle=flip180, system=system, return_gz=True,duration=2.51e-3,
     slice_thickness=slice_thickness,
                                  apodization=apodization,
                                time_bw_product=2)
""" READOUT
Readout gradients and related events
"""
delta_kx = 1 / fovx
delta_ky = 1 / fovy
k_width_x = Nx * delta_kx
k_width_y = Ny * delta_ky
gx = make_trapezoid(channel='x', system=system, flat_area=k_width_x, flat_time=readout_time)
adc = make_adc(num_samples=Nx, duration=gx.flat_time, delay=gx.rise_time)
""" PREPHASE AND REPHASE """
phase_areas = (np.arange(Ny) - (Ny / 2)) * delta_ky
gz_reph = make_trapezoid(channel='z', system=system, area=-gz90.area / 2,
                         duration=readout_time / 2)
gx_pre = make_trapezoid(channel='x', system=system, flat_area=k_width_x / 2,
                        flat_time=readout_time / 2)
gy_pre = make_trapezoid(channel='y', system=system, area=phase_areas[-1],
                        duration=readout_time / 2)
""" DELAYS
Echo time (TE) and repetition time (TR). Here, TE is broken down into `delay1` and `delay2`.
"""
delay1 -= calc_duration(rf180) / 2
delay1 = make_delay(delay1)
delay2 = tau - calc_duration(rf180) / 2
delay2 -= calc_duration(gx) / 2
delay2 = make_delay(delay2)
delay_TR = make_delay(delay_TR)
print(f'delay_1: {delay1}')
print(f'delay_2: {delay2}')
print(f'delay_TR: {delay_TR}')
""" CONSTRUCT SEQUENCE
Construct sequence for one phase encode and multiple slices
"""
# Prepare RF offsets. This is required for multi-slice acquisition
delta_z = n_slices * slice_gap
z = np.linspace((-delta_z / 2), (delta_z / 2), n_slices) + rf_offset
for k in range(nsa):  # Averages
  for j in range(n_slices):  # Slices
    # Apply RF offsets
    freq_offset = gz90.amplitude * z[j]
    rf90.freq_offset = freq_offset
    freq_offset = gz180.amplitude * z[j]
    rf180.freq_offset = freq_offset
    for i in range(Ny):  # Phase encodes
        seq.add_block(rf90, gz90)
        gy_pre = make_trapezoid(channel='y', system=system,
                 area=phase_areas[i], duration=readout_time / 2)
        seq.add_block(gx_pre, gy_pre, gz_reph)
```

```
        seq.add_block(delay1)
        seq.add_block(rf180, gz180)
        seq.add_block(delay2)
        seq.add_block(gx, adc)
        seq.add_block(delay_TR)
""" GENERATING `.SEQ` FILE """
# Prepare the sequence output for the scanner
seq.set_definition('FOV', [fovx, fovy, slice_thickness])
seq.set_definition('Name', 'SE')
seq.set_definition('Nx,Ny', [Nx,Ny])
seq.set_definition('flip', [rf_flip])
seq.set_definition('TR,TE', [TR,TE])
seq.set_definition('apodization', apodization)
seq.write(f'SE_{rf_flip}TE{int(TE*1000):03d}TR{int(TR*1000):04d}_ap{apodization}_Paper.seq')
```

## Geometry Generation

```
import trimesh
import numpy as np
import sys
import Geometry.from_stl_to_voxel as stl2vox
# Step 1: Create Two Concentric Disks (Cylinders)
# Disk 1 (radius 1, height 0.1)
disk_1 = trimesh.creation.cylinder(radius=10.0, height=1)
# Disk 2 (radius 0.5, height 0.1)
disk_2 = trimesh.creation.cylinder(radius=5.0, height=1)
# Exporting the mesh to STL format
disk_1.export('disk_out.stl')
disk_2.export('disk_in.stl')
resolution=[0.1,0.1,1]
stl_mesh, transp = stl2vox.load_and_translate_stl_to_positive_quadrant('disk_out.stl')
stl2vox.display_stl_with_matplotlib(stl_mesh)
#grid=stl2vox.VoxelGrid(resolution,np.array([0,0,0]),np.array([15,15,15]))
voxel_grid = stl2vox.create_voxel_grid(stl_mesh, resolution)
min_bound, max_bound = stl2vox.get_bounds(stl_mesh)
voxel_grid2 =stl2vox.VoxelGrid(resolution,min_bound,max_bound)
stl_mesh2, _ = stl2vox.load_and_translate_stl_to_positive_quadrant('disk_in.stl',transp)
voxel_grid2 = stl2vox.create_voxel_grid(stl_mesh2, resolution,voxel_grid2)
output=voxel_grid.data.astype(np.int32)
output[voxel_grid2.data]=2
# Save the voxel grid as a raw file
raw_filename = "2disk_grid.raw"
mhd_filename = "2disk_grid.mhd"
#save_voxel_grid_as_raw(voxel_grid, raw_filename)
stl2vox.save_voxel_data_as_raw_mhd(output, resolution,raw_filename,mhd_filename)
print(f"Voxelization complete. Voxel grid saved as {raw_filename}.")
```

For “import Geometry.from_stl_to_voxel as stl2vox” checkout the GitHub repository GAMS_public: `https://github.com/Morrighan89/GAMS_public`
